# Architected Inverse Nacre Hydrogels With High Strength and Crack‐Insensitive Toughness

**DOI:** 10.1002/advs.202523655

**Published:** 2026-05-21

**Authors:** Haidi Wu, Qin Su, Cheng Guan, Biwang Pan, Wenjie Hu, Jun Yan, Yifan Feng, Longcheng Tang, Xuewu Huang, Jiefeng Gao, Wancheng Gu

**Affiliations:** ^1^ School of Chemistry and Materials Yangzhou University Yangzhou Jiangsu China; ^2^ Key Laboratory of Organosilicon Chemistry and Material Technology of Ministry of Education Hangzhou Normal University Hangzhou China; ^3^ Testing Center Yangzhou University Yangzhou Jiangsu China

**Keywords:** mechanical properties, MXene, orientation, PVA hydrogels

## Abstract

The practical deployment of synthetic hydrogels in load‐bearing applications has long been hindered by their intrinsically low fracture toughness, poor fatigue resistance, and the persistent strength‐toughness trade‐off. Here, we propose an “inverse nacre” design principle that fundamentally overcomes these limitations by integrating hierarchical alignment of poly(vinyl alcohol) (PVA) chains and MXene nanosheets via a scalable thermo‐calendering process. In this inverted structure, a minimal quantity of aligned MXene nanosheets serves as continuous, high‐stiffness “bricks”, while the PVA hydrogel forms a ductile, energy‐dissipating “mortar”. This inversion introduces a dual‐function reinforcement mechanism: MXene nanosheets impart stiffness, crack‐bridging, and deflection capabilities, while simultaneously suppressing PVA crystallization, thereby promoting molecular alignment and enhancing deformability. The resulting cooperative architecture achieves an exceptional combination of tensile strength (63.48 MPa), work of fracture (54.79 MJ/m^3^), and record‐high fracture toughness (115.98 kJ/m^2^) without compromising hydrogel water content. Strikingly, the composite exhibits crack‐insensitive fracture behavior, where propagating cracks undergo extensive deflection and branching, activating an autonomous self‐preservation mechanism. This biomimetic strategy not only resolves the strength‐toughness paradox but also provides a generalizable route for designing structurally resilient soft materials with broad implications in biomedical and engineering applications.

## Graphical abstract

Hydrogels hold promise for biomedical and engineering applications, yet their practical use is fundamentally limited by the strength‐toughness trade‐off and poor fatigue resistance. In this study, a bioinspired inverse nacre architecture constructed via a scalable thermo‐calendering process is introduced, which overcomes these classic limitations through hierarchical alignment of poly(vinyl alcohol) chains and MXene nanosheets.

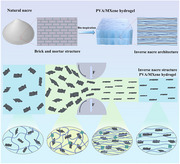

## 1 Introduction

Hydrogels, composed of 3D hydrophilic polymer networks capable of retaining substantial amounts of water, have garnered extensive attention in biomedical and soft materials science owing to their unique combination of biocompatibility, high water content, and tunable mechanical and physicochemical properties [1, 2, 3, 4]. These features make hydrogels promising candidates for diverse applications, including tissue engineering scaffolds, drug delivery systems, soft actuators, wound dressings, and wearable or implantable biosensors [5, 6, 7, 8]. Moreover, their structural and mechanical resemblance to biological soft tissues renders them ideal for interfacing with biological systems.

However, despite their versatility, the widespread deployment of hydrogels in mechanically demanding or load‐bearing applications remains fundamentally constrained by their inherently weak mechanical properties‐particularly low tensile strength, poor fracture toughness, and susceptibility to crack propagation [9]. These limitations often result from the homogeneous and isotropic nature of conventional polymer networks, which lack the hierarchical structures and energy dissipation mechanisms found in biological tissues. As a result, hydrogels often suffer catastrophic failure under mechanical stress, precluding their use in fields requiring durability, resilience, and resistance to fatigue or impact [10, 11].

To address these challenges, a multitude of strategies have been explored to improve the mechanical robustness of hydrogels [12, 13, 14, 15]. Bioinspired approaches, especially those mimicking the anisotropic architecture of natural load‐bearing tissues such as muscles, tendons, and ligaments, have shown considerable promise [16]. These strategies typically rely on constructing oriented microstructures through techniques like directional freezing [17], shear‐induced alignment [18], or external field‐assisted assembly [19]. Such structural anisotropy promotes chain orientation and interfacial interaction, enhancing tensile strength and resistance to crack initiation [20, 21]. Post‐processing methods such as thermal annealing or salting out further stabilize these aligned domains [22, 23]. In parallel, the incorporation of nanoscale reinforcements‐such as carbon nanotubes, graphene oxide, cellulose nanofibers, or aramid nanofibers‐has been investigated to improve stress transfer, crack arrest, and fatigue resistance [24, 25, 26, 27]. However, the effective translation of nanoscale reinforcement into macroscopic performance remains a major hurdle. Low filler content often results in insufficient reinforcement, while higher concentrations lead to undesirable agglomeration, phase separation, and pronounced embrittlement, ultimately undermining extensibility and fracture resistance [28, 29]. More critically, despite these efforts, achieving simultaneous high strength, high work of fracture, and large deformability in a single hydrogel system, a trilemma in polymer physics, remains an elusive goal [10]. Moreover, conventional nanofiller‐reinforced hydrogels rarely address crack‐growth insensitivity, an essential criterion for real‐world structural reliability.

Here, we report a biomimetic inverse nacre hydrogel that resolves the long‐standing strength‐work of fracture trade‐off in synthetic soft materials. By employing a scalable thermo‐calendering strategy, we induced the simultaneous orientation of MXene nanosheets and PVA chains, constructing a dual‐orientation architecture fundamentally distinct from classical nacre. In this configuration, trace aligned MXene nanosheets serve as continuous, high‐stiffness “bricks”, while the PVA hydrogel forms a ductile, energy‐dissipating “mortar”. Uniquely, the MXene phase not only provides reinforcement but also regulated PVA crystallization, suppressing excessive physical crosslinking and thereby enhancing molecular mobility and deformability. This counterintuitive dual role enabled the hydrogel to achieve an unprecedented mechanical synergy without compromising hydrogel water content, combining high strength (63.48 MPa), work of fracture (54.79 MJ/m^3^), and fracture energy (115.98 kJ/m^2^) with remarkable crack‐insensitive behavior and impact tolerance. Propagating cracks were forced into branching and deflection, activating an intrinsic self‐preservation mechanism rarely observed in hydrogels. Beyond establishing a generalizable principle for dual‐orientation engineering, this work advances the theoretical framework of architecture‐regulated energy dissipation and demonstrated a practical, scalable pathway toward ultra‐tough hydrogels for load‐bearing biomedicine and damage‐tolerant soft robotics.

## 2 Results and Discussion

### 2.1 The Fabrication of Dual‐Oriented Inverse Nacre Structure PM_x‐y_ Hydrogels

**FIGURE 1:**
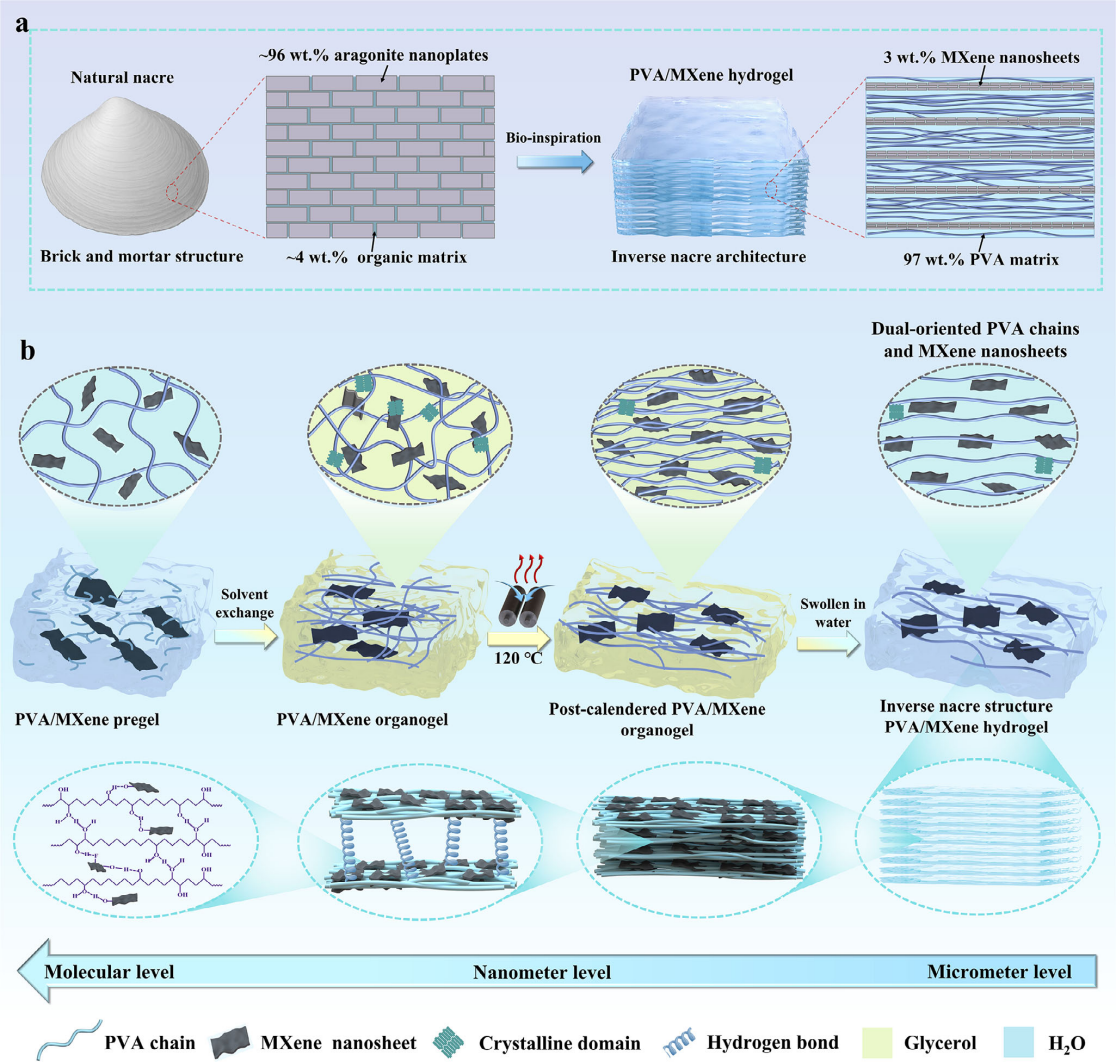
Bioinspired fabrication and architecture of dual‐oriented inverse nacre‐like PVA/MXene hydrogels. (a) Design concept: natural nacre with aragonite “bricks” in a biopolymer “mortar” (left) vs. the inverse nacre‐like hydrogel (right), where aligned MXene nanosheets act as “bricks” and oriented PVA lamellar domains serve as “mortar”; (b) Fabrication and structure evolution: solvent exchange induces PVA gelation and MXene immobilization, followed by thermo‐calendering that aligns both PVA chains and MXene nanosheets. The resulting hierarchical dual‐oriented structure is stabilized by multivalent MXene‐PVA hydrogen bonding and nanoconfinement.

Inspired by the hierarchical brick‐and‐mortar architecture of nacre [30, 31, 32, 33, 34], we devised a force‐field‐assisted confinement assembly strategy to engineer nanocomposite hydrogels with dual‐orientational order (Figure 1a). The central innovation lay in coupling solvent‐exchange‐induced kinetic trapping with thermo‐mechanical calendering, thereby achieving synchronized alignment of polymer chains and nanofillers across molecular and nanoscale levels. As illustrated in Figure 1b, the process began with MXene nanosheets (selected for their abundant oxygen‐containing functional groups and high surface hydrophilicity) serving as reinforcing agents within a poly(vinyl alcohol) (PVA) matrix (Figures S1 and S2). A homogeneous precursor solution was obtained by dispersing PVA in an aqueous MXene suspension, where strong MXene‐PVA hydrogen bonding and solvent‐polymer interactions suppressed premature chain aggregation and nanosheet restacking, ensuring structural uniformity.

A pivotal structural transition was induced by solvent exchange with glycerol, a poor solvent with significantly higher osmotic potential than water. The resulting osmotic gradient drove rapid glycerol infiltration and water efflux, leading to controlled dehydration of PVA chains. This solvent‐driven confinement triggered cooperative chain collapse and aggregation, generating semicrystalline domains that acted as robust physical cross‐links and promoted organogel formation [35, 36]. Concurrently, MXene nanosheets became kinetically immobilized within the evolving polymer network through multivalent hydrogen bonding and steric confinement, achieving homogeneous nanoscale dispersion without macroscopic phase separation, a longstanding challenge in nanocomposite hydrogel engineering. The resulting organogel exhibited enhanced thermal stability and mechanical cohesion, prerequisites for subsequent structural programming. Under coupled thermal and mechanical fields during thermo‐calendering, the organogel underwent multiscale structural reorganization. Thermally enhanced chain mobility facilitated shear‐induced stacking of PVA into ordered lamellae, while stress‐mediated reorientation aligned MXene nanosheets parallel to the shear plane, forming periodically intercalated layers. This dual‐oriented hierarchical structure was stabilized by topological confinement and multivalent interfacial bonding, enabling the retention of the laminate structure even after hydration and thereby overcoming the prevalent issue of structural relaxation in anisotropic hydrogels [37].

The final material embodied an inverse nacre architecture: unlike classical nacre dominated by mineral platelets in a soft matrix, here a sparse yet continuous network of aligned MXene nanosheets (<5 wt.%) functioned as the reinforcing “bricks”, while the oriented semi‐crystalline PVA domains provided the ductile, energy‐dissipating “mortar”. This inversion of the classical bioinspired structure established a dual‐percolating architecture that synergistically enhanced mechanical strength and fracture toughness through crack deflection, interfacial load transfer, and reversible bond reorganization. More broadly, this methodology established a generalizable platform for constructing hierarchically ordered soft materials through kinetic trapping and field‐directed assembly, opening avenues for high‐performance nanocomposites with programmable microstructures. For clarity, the PVA/MXene/glycerol organogels were designated as PM_x_O organogels, and calendered hydrogels were designated PM_x‐y_ hydrogels, where x represented MXene content relative to PVA, and y denoted the roller nip spacing during calendering. Control samples, prepared without calendering by direct hydration of the PM_x_O organogels, were labeled PM_x_ hydrogels.

### 2.2 Morphology and Crystalline Structural Analysis of the PM_x‐y_ Hydrogels

**FIGURE 2:**
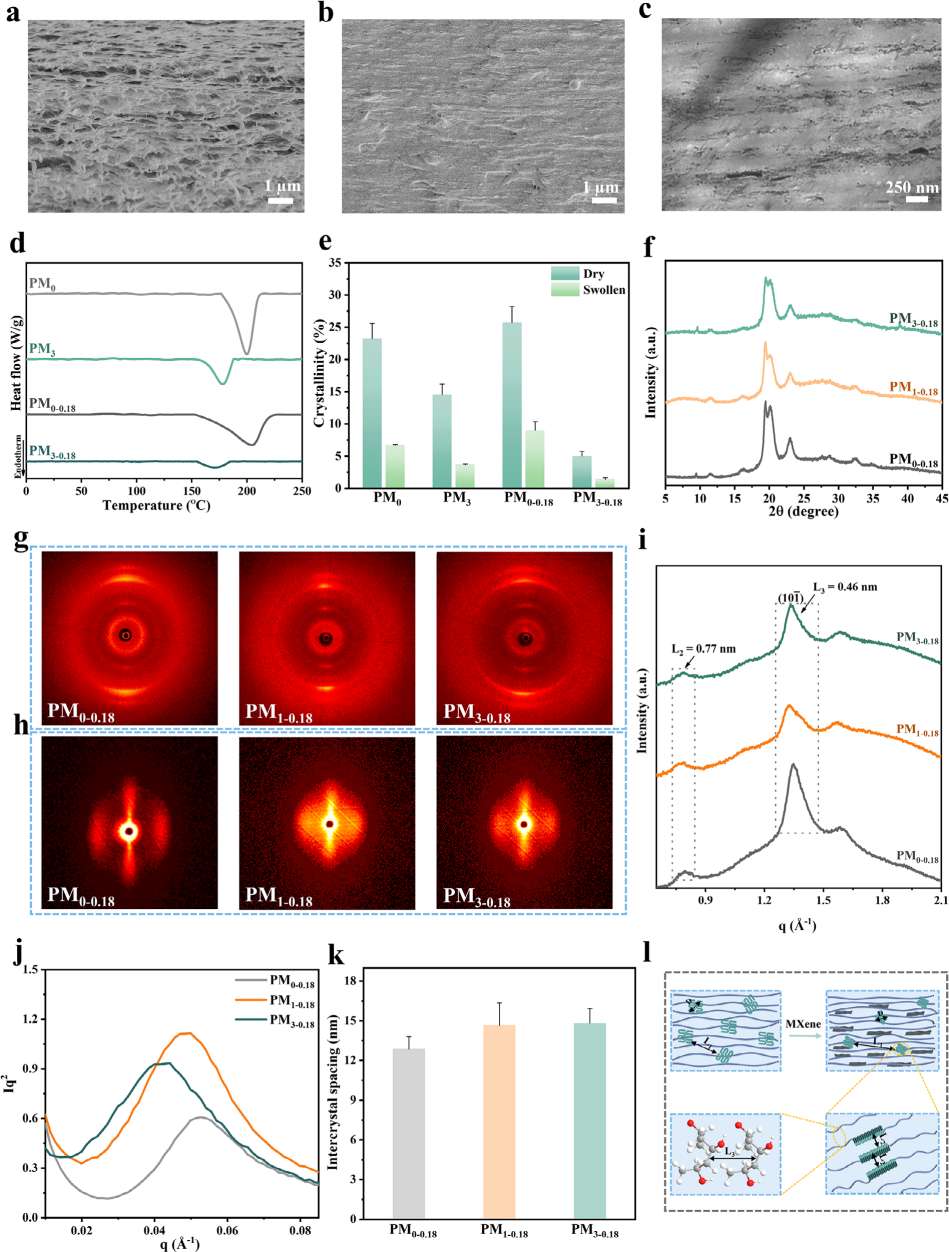
Morphology and crystalline structural analysis of the PVA hydrogels. (a,b) SEM images of the PM_0‐0.18_ hydrogel (a) and the PM_3‐0.18_ hydrogel (b); (c) TEM image of the PM_3‐0.18_ hydrogel; (d,e) DSC thermographs of different PM_x_ and PM_x‐0.18_ hydrogels (d) and the corresponding crystallinity in dry and swollen states of these hydrogels (e); (f) XRD spectra of PM_x‐0.18_ hydrogels; (g,h) 2D WAXS patterns (g) and SAXS patterns (h) of PM_x‐0.18_ hydrogels; (i) The scattering intensity vs. scattering vector (q) curves for WAXS curves of PM_x‐0.18_ hydrogels; (j) The corresponding scattering intensity vs. q curves for SAXS curves of PM_x‐0.18_ hydrogels; (k) The calculated intercrystal spacing of PM_x‐0.18_ hydrogels by SAXS; (l) Schematic diagram of intercrystal spacing (L_1_), crystal dimension (D), interlamellar spacing (L_2_), intermolecular spacing (L_3_) of the PM_x‐0.18_ hydrogels.

Subsequent microstructural analysis of the PM_x_ and PM_x‐0.18_ hydrogels via scanning electron microscopy (SEM) revealed a sophisticated architectural evolution driven by the synergistic interplay between thermo‐mechanical calendering and MXene incorporation. The control PM_0_ hydrogel, devoid of MXene and calendering, displayed a conventional isotropic and porous network morphology (pore size ∼122 nm, Figure S3a). Incorporation of a minimal quantity of MXene nanosheets (PM_3_ hydrogel) induced a substantial architectural densification, with porosity becoming indiscernible due to nanoconfinement effects and enhanced intermolecular interactions between MXene surfaces and PVA chains (Figure S3b). Calendering processing triggered a fundamental structural transition from isotropy to anisotropy. The unreinforced PM_0‐0.18_ hydrogel developed a unidirectional lamellar morphology under shear and compression; however, insufficient interlamellar cohesion resulted in structural discontinuities and microvoids (Figure 2a; Figure S4a). In stark contrast, the PM_3‐0.18_ hydrogel exhibited a highly integrated, layered nanocomposite architecture with no detectable defects, indicating effective polymer‐nanofiller integration and complete pore‐filling during deformation (Figure 2b; Figure S4b). This suggested that MXene nanosheets not only functioned as a conventional reinforcement but also as an active templating agent that directed the reorganization of the PVA matrix under thermo‐mechanical fields. Key to this structural achievement was the shear‐induced preferential alignment of MXene nanosheets (“bricks”) within a continuous, oriented PVA matrix (“mortar”), collectively forming a bioinspired inverse nacre‐like architecture. Transmission electron microscopy (TEM) further confirmed this hierarchical organization, showing MXene nanosheets aligned parallel to the PVA lamellae and seamlessly integrated between adjacent polymer domains (Figure 2c). However, exceeding the optimal MXene concentration resulted in nanosheet aggregation and structure nonuniformity (e.g., PM_5‐0.18_ hydrogel in Figure S4c), highlighting the criticality of interfacial compatibility and dispersion homogeneity for achieving structural uniformity and mechanical robustness. This systematic microstructural investigation substantiated the efficacy of our dual‐orientation strategy in fabricating hierarchically ordered hydrogels with enhanced structural integrity.

The presence of MXene nanosheets profoundly influenced the crystalline architecture and supramolecular ordering, as elucidated by thermal and diffraction analyses. Differential scanning calorimetry (DSC) of the unreinforced PM_0_ hydrogel showed a distinct endothermic melting peak between 180°C–220°C [38], corresponding to a dry‐state crystallinity of 23.19% (Figure 2d,e). Thermo‐calendering further elevated the crystallinity to 25.67% in PM_0‐0.18_ hydrogel, indicative of stress‐induced crystallization under coupled thermal‐mechanical activation. Introduction of MXene nanosheets significantly suppressed overall crystallinity, which decreased to 14.47% in the uncalendered PM_3_ hydrogel. This reduction was attributed to a nanoconfinement mechanism: the uniformly dispersed MXene occupied intermolecular spaces, thereby impeding the supramolecular reorganization necessary for nucleation and crystal growth. This inhibitory effect was further accentuated by calendering, with crystallinity demonstrating a strong negative correlation with MXene loading. The PM_3‐0.18_ hydrogel exhibited a crystallinity of merely 4.28%, and crystalline domains became virtually undetectable in the PM_5‐0.18_ hydrogel at 5 wt.% MXene (Figure S5). X‐ray diffraction (XRD) analyses corroborated these observations: the diffraction intensities at 2θ = 19.8° and 23.0°, corresponding to the (10$\bar{1}$), (101), and (200) planes of semicrystalline PVA [39], progressively diminished with increasing MXene content (Figure 2f; Figures S6 and S7). These results confirmed that interfacial hydrogen bonding between MXene surface functional groups and PVA chains, combined with spatial confinement imposed by calendering, effectively disrupted long‐range crystalline order.

The anisotropic structural features of the calendered PM_x‐0.18_ hydrogels were quantitatively resolved through combined wide‐angle and small‐angle x‐ray scattering (WAXS/SAXS). Both 2D WAXS and SAXS patterns exhibited pronounced anisotropic diffraction arcs (Figure 2g,h), consistent with the aligned lamellar morphology observed via electron microscopy [40]. Azimuthal integration of the WAXS patterns yielded intensity maxima at 0° and 180°, confirming a high degree of uniaxial orientation within the nanocomposite (Figure S8a). The Herman's orientation factor systematically decreased from 0.87 for the PM_0‐0.18_ hydrogel to 0.77 for the PM_5‐0.18_ hydrogel (Figure S8b), indicating that increased MXene content progressively restricted macromolecular alignment. This reduction in orientational order arose from two synergistic mechanisms: (1) the incorporation of rigid MXene nanosheets enhanced network stiffness, thereby limiting large‐strain deformability and chain mobility during calendering; and (2) strong interfacial interactions, evidenced by a significant redshift of the O─H stretching vibration in FTIR spectra from 3332.4 cm^−1^ (PM_0‐0.18_ hydrogel) to 3211.2 cm^−1^ (PM_3‐0.18_ hydrogel), restricted the directional reorganization of polymer chains (Figure S9) [41]. These interfacial interactions were further quantified by an increase in glass transition temperature (Tg) from 100.31°C (PM_0‐0.18_ hydrogel) to 102.04°C (PM_3‐0.18_ hydrogel, Figure S10), confirming constrained segmental mobility within the nanocomposite hydrogels.

WAXS profiles indicated a reduction in the (10$\bar{1}$) reflection intensity, while the interlamellar (0.77 nm) and intermolecular (0.46 nm) spacings remained unaffected (Figure 2i; Figure S11), suggesting that MXene primarily inhibited crystal growth without altering unit cell geometry [42]. Scherrer analysis revealed a gradual decrease in crystallite size from 6.60 nm (PM_0‐0.18_ hydrogel) to 6.33 nm (PM_3‐0.18_ hydrogel) with increasing MXene content (Figure S12), affirming restricted crystal dimension. SAXS profiles exhibited a shift of the scattering peak toward lower q‐values (Figure 2j; Figure S13), indicating an increase in the long‐period spacing (L_1_) from 12.87 nm (PM_0‐0.18_ hydrogel) to 14.81 nm (PM_3‐0.18_ hydrogel, Figure 2k) [43]. This increase reflected the expansion of interlamellar amorphous regions due to MXene intercalation and consequent disruption of crystalline packing, underscoring the role of MXene as a multifunctional modulator of crystallinity and nanoscale organization in these hierarchically structured hydrogels (Figure 2l).

### 2.3 Mechanical Properties of the PM_x‐y_ Hydrogels

**FIGURE 3:**
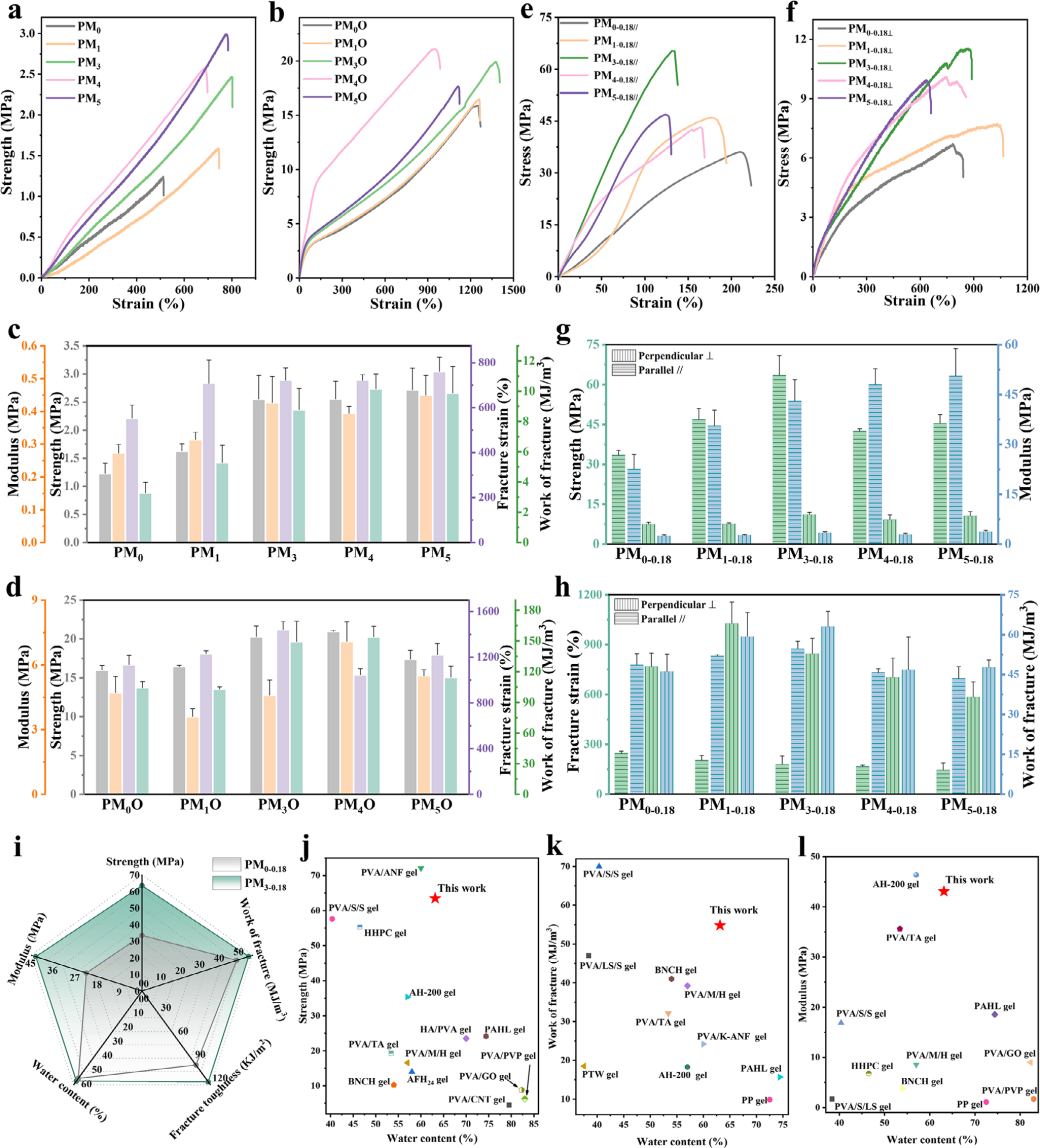
Mechanical properties of dual‐oriented PM_x‐y_ hydrogels. (a,b) Tensile stress–strain curves of the PM_x_ hydrogels (a) and PM_x_O organogels (b) with varying MXene contents; (c,d) Summary of tensile strength, fracture strain, modulus, and work of fracture for the PM_x_ hydrogels (c) and PM_x_O organogels (d); (e,f) Tensile stress–strain curves of the PM_x‐0.18_ hydrogels tested along the orientation‐parallel (e) and orientation‐perpendicular (f) directions; (g,h) Corresponding comparisons of tensile strength and modulus (g), as well as fracture strain and work of fracture (h) between the two directions; (i) Direct comparison of overall mechanical performance between the isotropic PM_0‐0.18_ hydrogel and the dual‐oriented PM_3‐0.18_ hydrogel; (j–l) Ashby plots of tensile strength vs. water content (j), work of fracture vs. water content (k), and modulus vs. water content (l) of the PM_3‐0.18_ hydrogel with other reported high strength anisotropic PVA hydrogels.

To unravel the structure‐property correlation of the inverse nacre‐inspired architecture, the macroscopic mechanical behavior of the PM_x‐y_ hydrogels was systematically examined. As shown in Figure 3a, the pristine PVA hydrogel (PM_0_) displayed a typical trade‐off between strength and extensibility, with a tensile strength of 1.2 MPa and a fracture strain of 553.7% (Figure 3c). This well‐known paradox originated from the limited ability of conventional PVA hydrogels to simultaneously sustain load transfer and chain mobility. Remarkably, the incorporation of a small fraction of MXene nanosheets disrupted this limitation by enabling a concurrent enhancement in both strength and ductility. For instance, the PM_1_ hydrogel achieved a tensile strength of 1.6 MPa, a fracture strain of 708.7%, and the PM_3_ hydrogel achieved a tensile strength of 2.6 MPa, an elongation at break of 722.6%, and a work of fracture of 8.8 MJ/m^3^. Mechanistically, MXene nanosheets acted as multifunctional regulators: while their incorporation reduced the crystalline fraction of PVA, thereby improving chain extensibility, this potential loss in strength was offset by the nanosheets’ intrinsic rigidity and the formation of robust interfacial hydrogen‐bonding interactions with PVA chains, which facilitated efficient stress transfer. Further enhancement was achieved by solvent exchange with glycerol, which transformed PM_x_ hydrogels into PM_x_O organogels (Figure 3b,d). In this case, glycerol molecules as a poor solvent promoted chain aggregation and densified entanglement networks, while simultaneously strengthening inter‐chain hydrogen bonding. As a result, the PM_3_O organogel demonstrated a large tensile strength of 20.3 MPa, fracture strain of 1442.2%, and work of fracture of 149.5 MJ/m^3^. The excellent mechanical properties as well as thermal stability provided an ideal precursor for subsequent thermo‐calendering.

Thermo‐calendering introduced a second level of structural control by imposing shear and compression, which induced concurrent alignment of MXene nanosheets and PVA chains. The resulting dual‐oriented architecture yielded a dramatic leap in performance. Along the alignment direction, the PM_0‐0.18_ hydrogel displayed 33.5 MPa strength and 246.5% fracture strain, confirming the essential role of molecular orientation in reinforcing polymer networks. With 3 wt.% MXene incorporation, the PM_3‐0.18_ hydrogel reached optimal properties: 63.48 MPa tensile strength, 43.1 MPa modulus, and 54.79 MJ/m^3^ work of fracture (Figure 3e–g), achieving both strengthening and toughening. These values surpassed most reported anisotropic hydrogels, highlighting the capacity of the inverse nacre structure to decouple strength and ductility. However, further increasing MXene to 4 and 5 wt.% reduced mechanical performance due to nanosheet aggregation and stress concentration. Notably, the dual‐oriented hydrogels retained improvements in the transverse direction as well. Even perpendicular to the primary alignment direction, the PM_3‐0.18⊥_ hydrogel exhibited exceptional mechanical properties, with a strength of 11.1 MPa and a work of fracture of 55.1 MJ/m^3^, a value that surpassed the work of fracture of PM_3‐0.18//_ (Figure 3f–h). Furthermore, both the fracture strain and work of fracture in this perpendicular orientation exceeded those of the PM_0‐0.18_ hydrogel tested along its alignment axis. This unusual mechanical characteristic highlighted a fundamental advantage of the inverse nacre architecture: the continuous, aligned MXene network and its strong interfacial bonding with the polymer matrix facilitated efficient stress transfer and energy dissipation in multiple directions, effectively defying the conventional trade‐offs between anisotropy and multi‐axial mechanical performance.

Mechanical properties could be further tuned by adjusting the roller nip during calendering (Figure S14). Narrower nips intensify compressive and shear stresses, leading to greater molecular alignment and densification. Accordingly, as the roller nip spacing decreased from 0.5 to 0.18 mm, the tensile strength increased systematically from 11.5 MPa (PM_3‐0.5_) to 63.48 MPa (PM_3‐0.18_), while the fracture strain correspondingly decreased from 411.84% to 180.34%. Importantly, water content was minimally affected by MXene incorporation (Figure S15). Even at 3 wt.% MXene, water content remained 63.1%, thereby resolving the typical incompatibility between mechanical robustness and high hydration in hydrogels. We also investigated the swelling kinetics of the PM_3‐0.18_ hydrogel. Specifically, we conducted swelling experiments by immersing freeze‐dried PM_3‐0.18_ hydrogel samples in deionized water at about 20°C and measuring the weight increase at predetermined time intervals until equilibrium was reached. As shown in Figure S16, the PM_3‐0.18_ hydrogel exhibited typical swelling kinetics characterized by a rapid initial water uptake during the first 60 min, followed by gradual deceleration and eventual plateau after approximately 3 h. And the equilibrium swelling ratio reached approximately 167.4% after 24 h, which corresponded to an equilibrium water content of about 62.6%, consistent with the original water content of the as‐prepared hydrogel. This indicated that the hydrogel maintained its hydration state without excessive swelling or dissolution. The PM_3‐0.18_ hydrogel exhibited significant overall improvements in mechanical properties compared with the PM_0‐0.18_ hydrogel (Figure 3i), highlighting the superiority of the inverse nacre‐like structure. In addition, to benchmark the performance of the optimized PM_3‐0.18_ hydrogel, we constructed Ashby plots comparing strength, work of fracture, modulus, and water content with previously reported anisotropic PVA hydrogels (Figure 3j–l). The PM_3‐0.18_ hydrogel uniquely integrated high tensile strength (>63 MPa), large work of fracture (>54 MJ/m^3^), and high water content (>63%), establishing it as one of the best‐performing hydrogels by comparison with reported tough PVA hydrogel materials (Table S1). More fundamentally, these results demonstrated that the inverse nacre strategy enabled the decoupling of strength, work of fracture, and hydration‐properties traditionally constrained by mutual trade‐offs.

Beyond static mechanical properties, the dynamic viscoelastic behavior further confirmed the reinforcing and toughening effect of MXene incorporation. Frequency‐sweep tests demonstrated that all PM hydrogels exhibited elastic‐dominant responses [38], with storage modulus (*G*′) consistently exceeding loss modulus (*G*″) across the measured frequency range (Figure S17a,b). Notably, *G*′ values for the PM_3_ and PM_3‐0.18_ hydrogels were substantially higher than those of the MXene‐free PM_0_ and PM_0‐0.18_ hydrogels, demonstrating the contribution of MXene nanosheets to network elasticity. The strain‐dependent evolution of *G*′ and *G*″ further revealed critical insights into the deformation tolerance of the dual‐oriented inverse nacre hydrogels (Figure S17c,d). At small strains, the systems maintained a pronounced elastic character (*G*′>*G*″); however, with increasing strain, *G*′ progressively decayed until reaching an intersection point with *G*″, which marked the onset of nonlinear viscoelastic behavior and the breakdown of the elastic network [36]. Strikingly, the critical strain corresponding to this crossover shifted to higher values upon MXene incorporation. This shift indicated an enhanced ability of the PM_3_ and PM_3‐0.18_ hydrogels to withstand large‐amplitude deformations before network yielding, thereby reflecting superior mechanical resilience.

These macroscopic improvements originated from the synergistic interplay between molecular interactions and mesoscale structure. On the molecular level, abundant surface functionalities on MXene nanosheets formed robust yet reversible hydrogen bonds with the hydroxyl groups of PVA chains. These sacrificial interactions could reversibly break and reform under deformation, dissipating mechanical energy and mitigating local stress concentration. At the mesoscale, the dual‐oriented nacre‐like architecture enabled efficient load redistribution: aligned MXene nanosheets and PVA fibrils acted as stress‐bearing “bricks”, while amorphous chain networks served as energy‐dissipating “mortar”. Taken together, the integrated static and dynamic analyses established a clear structure‐performance‐mechanism correlation: the unique dual‐orientation architecture, reinforced by MXene‐PVA supramolecular interactions, enabled hydrogels that simultaneously achieve high strength, work of fracture, and resilience. This synergistic mechanism provided a generalizable design principle for engineering bioinspired polymer composites with damage tolerance and multifunctionality.

### 2.4 Fatigue Resistance of the PM_x‐y_ Hydrogels

To further evaluate the fatigue resistance and fracture behavior of the PM_x‐0.18_ hydrogels, cyclic tensile tests were performed. The PM_3‐0.18_ hydrogel displayed pronounced hysteresis during the first loading‐unloading cycle, with a tensile strength of 29.66 MPa and an energy dissipation of 4.95 MJ/m^3^, evidencing its exceptional capacity for energy absorption (Figure S18a,b). Although both strength and dissipated energy gradually decreased upon cycling, the hydrogel retained a residual strength of 16.94 MPa and a dissipation of 50.03 kJ/m^3^ after 1000 cycles, underscoring its high fatigue stability. Moreover, energy dissipation was strongly strain‐dependent: while modest at 25% strain (dissipated energy of 0.67 MJ/m^3^, energy dissipation coefficient of 52.38%), it surged beyond 75% strain, reaching 18.14 MJ/m^3^ at 175% strain with an energy dissipation coefficient of 71.90% (Figure S18c,d). These results highlighted the hydrogel's strain‐adaptive energy dissipation, arising from its dual‐oriented inverse nacre‐like architecture.

**FIGURE 4:**
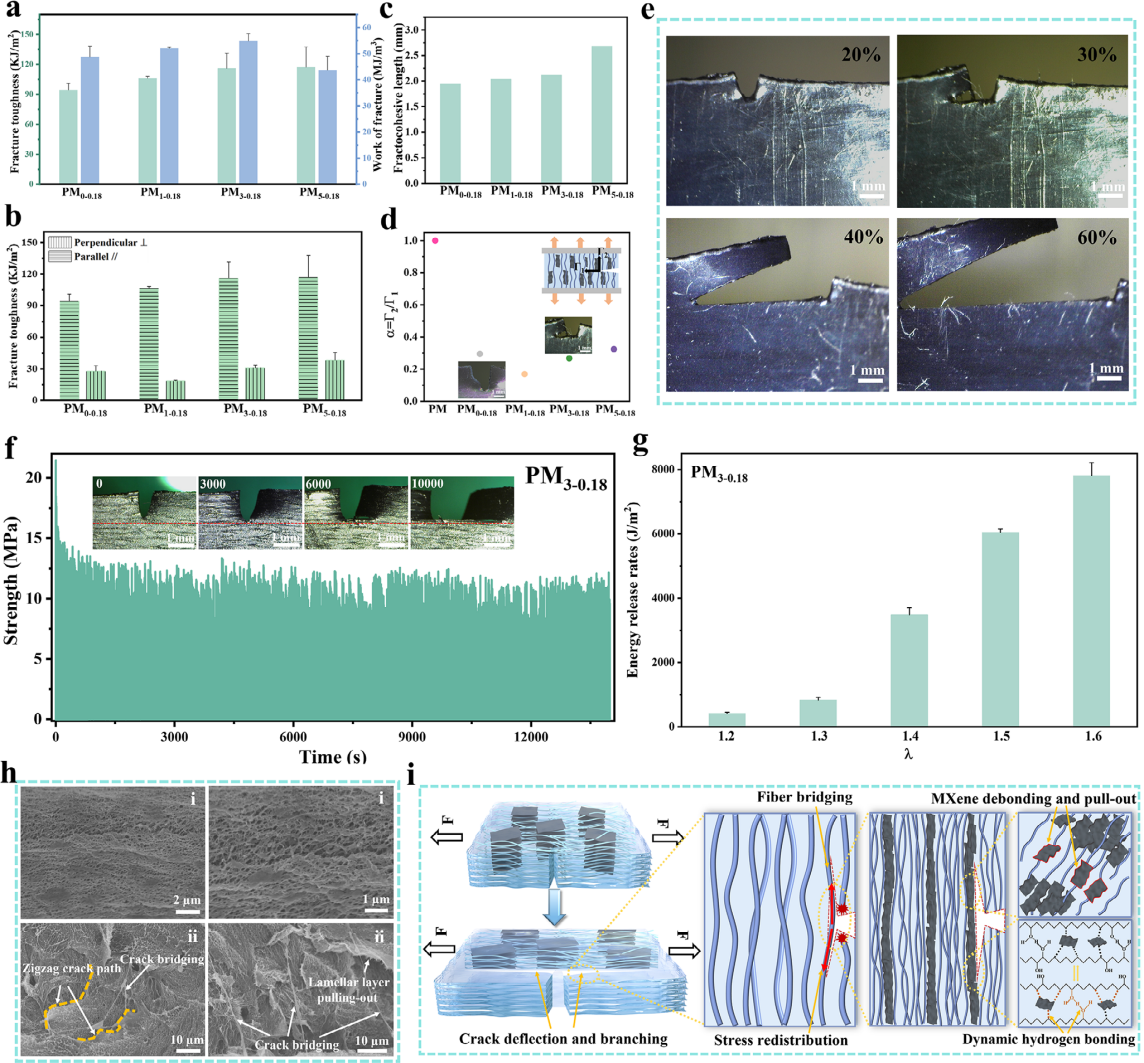
Fatigue resistance and anisotropic crack‐propagation behavior of the PM_x‐0.18_ hydrogels. (a) Comparison of fracture toughness and work of fracture among the PM_x‐0.18_ hydrogels; (b) Orientation‐dependent fracture toughness values parallel (Γ_1_) and perpendicular (Γ_2_) to the alignment direction; (c) Fractocohesive length (Γ/W, fracture toughness divided by work of fracture) of the PM_x‐0.18_ hydrogels; (d) Anisotropy ratio of fracture toughness (α = Γ_2_/Γ_1_), with inset images showing distinct crack propagation modes of the PM_0‐0.18_ and PM_3‐0.18_ hydrogels; (e) Optical images of strain‐dependent crack trajectories in the PM_3‐0.18_ hydrogel, demonstrating spontaneous deflection and branching; (f) Strength of the precut PM_3‐0.18_ hydrogel during 10 000 loading‐unloading cycles at 30% strain, with insets showing notch evolution after different cycle numbers; (g) Energy release rate as a function of applied strain for the PM_3‐0.18_ hydrogel; (h) SEM micrographs of the fracture cross‐section of the PM_0‐0.18_ hydrogel (h_i_) and PM_3‐0.18_ hydrogel (h_ii_); (i) Schematic illustration of the hierarchical fatigue‐ and crack‐resistance mechanisms enabled by the dual‐oriented inverse nacre‐like architecture.

The dual‐oriented inverse nacre‐like structure can also improve the fracture toughness of the PM_x‐y_ hydrogels (Figure 4a–c). Along the parallel orientation, the fracture toughness rose from 94.42 kJ/m^2^ (the PM_0‐0.18_ hydrogel) to 106.09 (PM_1‐0.18_ hydrogel) and 115.98 kJ/m^2^ (PM_3‐0.18_ hydrogel), respectively, surpassing most previously reported anisotropic PVA hydrogels (Figure S19 and Table S1). Perpendicular fracture toughness also improved, from 27.87 (PM_0‐0.18_ hydrogel) to 31.04 kJ/m^2^ (PM_3‐0.18_ hydrogel). Simultaneously, the fractocohesive length [44], a descriptor of flaw sensitivity defined as fracture toughness divided by work of fracture increased from 1.94 mm (PM_0‐0.18_ hydrogel) to 2.68 mm (PM_5‐0.18_ hydrogel), indicating a transition toward reduced crack sensitivity and enhanced defect tolerance [45].

The anisotropy of fracture toughness further dictated crack propagation modes. The dimensionless parameter α = Γ_2_/Γ_1_ (ratio of perpendicular to parallel fracture toughness) was closely associated with crack deflection [46]. Specifically, the PM_0‐0.18_ hydrogel (α = 0.30) exhibited straight crack propagation without observable deflection (Figure S20), the dual‐oriented PM_3‐0.18_ hydrogel (α = 0.26, below the critical threshold) underwent spontaneous crack deflection and branching under loading (Figure 4d), effectively dissipating fracture energy and protecting the structural integrity of the material. Even under 60% strain, cracks deviated from the notch direction, dispersing into multiple branches that redistributed stresses and delayed final failure, a hallmark of intrinsic flaw tolerance and self‐protection (Figure 4e). Remarkably, under notched conditions, the PM_3‐0.18_ hydrogel endured 10 000 loading‐unloading cycles at 30% strain with negligible crack growth (Figure 4f). Notably, the energy release rate of the PM_3‐0.18_ hydrogel underwent a substantial increase with escalating tensile strain, surging from 415.85 J/m^2^ at λ = 1.2 to 7813.09 J/m^2^ at λ = 1.6, far surpassing the fatigue threshold of the PM_0‐0.18_ hydrogel (2013.83 J/m^2^, Figure 4g; Figure S21). The exceptional mechanical synergy of the PM_3‐0.18_ is fundamentally rooted in the multivalent interfacial interactions between MXene and the PVA matrix. The abundant oxygen‐containing groups on MXene nanosheets formed robust hydrogen bonds with PVA hydroxyls. These interactions served a dual purpose: they acted as reversible sacrificial bonds for efficient energy dissipation during deformation, while simultaneously restricting PVA chain mobility to modulate crystallinity and enhance interfacial stress transfer. Crucially, this strong interfacial affinity ensured long‐term composite compatibility and structural stability. The hydrogen‐bonding network effectively immobilizes MXene nanosheets within the matrix, preventing aggregation or leaching and preserving the inverse nacre architecture under hydrated conditions. Furthermore, the reversible nature of these interfacial bonds enabled continuous energy dissipation without cumulative damage, underpinning the hydrogel's remarkable fatigue resistance over 10 000 cycles. Thus, the MXene‐PVA interface not only drove the initial mechanical enhancement but also guaranteed the durability and environmental reliability of the composite.

Scanning electron microscopy (SEM) of fracture cross‐sections unveiled a hierarchical damage‐tolerance architecture within the PM_3‐0.18_ hydrogel, markedly distinct from the relatively clean fracture observed in the unreinforced PM_0‐0.18_ hydrogel (Figure 4h). The nanocomposite exhibited multi‐mode failure mechanisms including lamellar pull‐out, interlayer delamination, extensive crack bridging, and highly tortuous propagation paths indicative of intensive energy dissipation [42, 47]. These features collectively underscore an innate crack‐resistant design rooted in the synergistic multiscale hierarchy of the inverse nacre architecture (Figure 4i). At the microscale, when a crack propagated along its initial trajectory, the oriented fiber bundles composed of aligned PVA chains facilitated the redistribution of stress to adjacent fibrils via interfacial interactions (hydrogen bonding), thereby alleviating excessive stress concentration at the crack tip. Some elastic fibrils could bridge across incipient microcracks, effectively inhibiting further crack opening and thereby delaying crack propagation. Additionally, the periodic alternation of rigid MXene nanosheets and ductile PVA lamellae promoted controlled interfacial debonding and initiated MXene pull‐out upon loading, effectively dissipating energy through frictional sliding and nano‐reinforcer extraction. Concurrently, the high stiffness of MXene induced crack‐tip blunting and forced deflection along weak interfaces, resulting in meandering, zigzag crack trajectories, and autonomous branching that dramatically extend the total fracture path. At the molecular level, the dynamic yet robust hydrogen‐bonding network (both at the MXene‐PVA interface and within the PVA matrix) facilitated efficient stress transfer and enabled reversible rupture‐reformation cycles under cyclic loads, thereby mitigating damage accumulation and enhancing fatigue life. These complementary energy‐dissipation mechanisms, operating coherently across nano‐ to micro‐scales, established a continuous stress‐redistribution network that effectively suppressed catastrophic crack propagation [48, 49]. The resultant material exhibited unprecedented autonomous crack inhibition and exceptional fatigue tolerance, positioning it as a benchmark among synthetic hydrogels for applications demanding fracture resistance and mechanical durability.

### 2.5 Applications of the PM_3‐0.18_ Hydrogels

In practical applications, hydrogels are frequently subjected to aqueous and mechanically challenging environments, making swelling resistance, structural integrity, and damage tolerance essential for long‐term functionality. To evaluate the environmental reliability of our high‐performance hydrogels, we systematically assessed their chemical, tear, and impact resistance, all of which underscore the fundamental superiority of the inverse nacre architecture.

**FIGURE 5:**
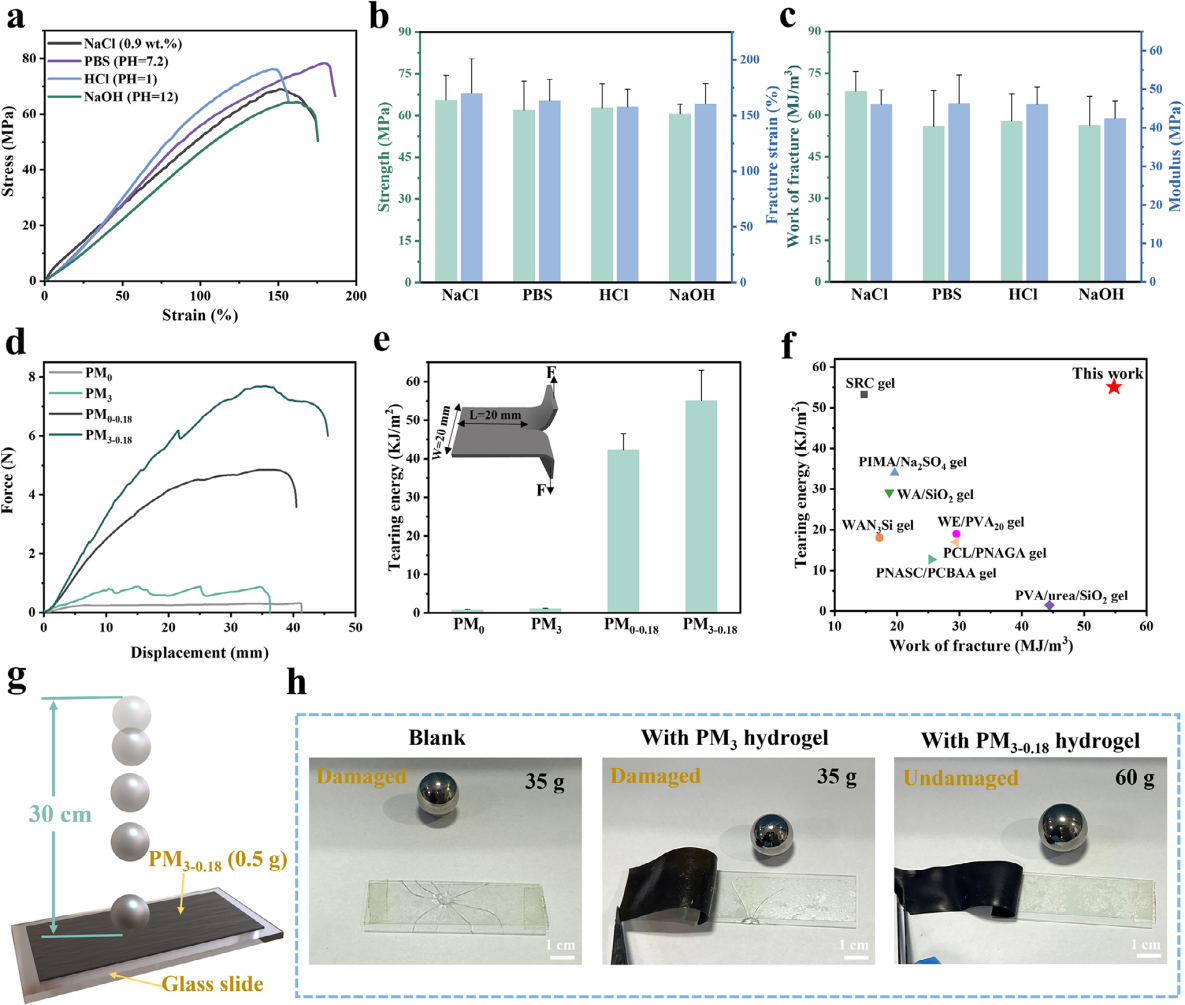
Swelling, tear, and impact resistance of the PM_3‐0.18_ hydrogels. (a–c) Retained tensile strength, fracture strain, modulus, and work of fracture of the PM_3‐0.18_ hydrogels after 7‐day immersion in NaCl, PBS, HCl, and NaOH solutions, demonstrating exceptional anti‐swelling stability; (d,e) Tear resistance of the PM_x_ and PM_x‐0.18_ hydrogels, showing progressive improvement from MXene incorporation and thermo‐calendering; (f) Comparison plots of tearing energy vs. work of fracture of the PM_3‐0.18_ hydrogel with other high strength hydrogels reported before; (g,h) Drop‐ball impact protection tests, where a thin PM_3‐0.18_ hydrogel layer prevented glass fracture under severe impact.

The PM_3‐0.18_ hydrogel exhibited exceptional chemical stability and anti‐swelling behavior when immersed for 7 days in physiological saline, PBS, and aggressive media (HCl, pH = 1; NaOH, pH = 13). Under all conditions, the PM_3‐0.18_ hydrogel retained nearly identical tensile strength, fracture strain, and work of fracture (Figure 5a–c). This stability originated from the densely aligned, dual‐oriented microstructure: MXene nanosheets interlock with oriented PVA chains to form a compact, hierarchically ordered network that restricted polymer chain mobility and counteracted osmotic‐driven water ingress, thereby preserving mechanical properties even under extreme chemical exposure.

Tear resistance tests further revealed the structural robustness imparted by the inverse nacre design. The tearing energy increased from 0.82 kJ/m^2^ for the pure PVA hydrogel (PM_0_ hydrogel) to 1.15 kJ/m^2^ for the uncalendered PM_3_ hydrogel. Most notably, thermo‐calendering amplified this value by over 40 times, reaching 55.11 kJ/m^2^ for the PM_3‐0.18_ hydrogel (Figure 5d,e). To systematically evaluate the anisotropic tear resistance, we conducted tearing tests at three orientations relative to the alignment direction: 0° (parallel), 45°, and 90° (perpendicular). As shown in Figure S22, the tearing energy exhibited a pronounced orientation dependence, increasing progressively from 27.84 kJ/m^2^ at 90° to 39.97 kJ/m^2^ at 45°, and reaching 55.11 kJ/m^2^ at 0°. This approximately twofold enhancement from the parallel to perpendicular orientation demonstrates the significant anisotropy in tear resistance imparted by the inverse nacre architecture. The corresponding crack propagation paths, also presented in Figure S23, revealed distinct fracture behaviors depending on the testing orientation. When the tearing direction was parallel to the alignment direction (at 0°), the crack propagated in a relatively straight manner with minimal deflection, following the path of least resistance along the oriented lamellar interfaces. The crack advanced steadily along the initial direction until complete sample failure occurred. In contrast, when the tearing direction was oriented at 45° or 90° relative to the alignment direction, the crack exhibited significant deflection and meandering behavior. The crack no longer followed a straight trajectory, instead, it propagated along a tortuous path. Notably, as the testing angle increased from 45° to 90°, the degree of crack deflection became more pronounced, with the crack deviating further from the initial notch direction. The observed orientation‐dependent tearing behavior could be rationalized by considering the hierarchical structure of the inverse nacre architecture. When the tearing force was applied perpendicular to the alignment direction (90°), the crack must propagate across the oriented MXene nanosheets and PVA lamellae, repeatedly rupturing through the stiff MXene “bricks” and strong interfacial hydrogen bonds, thereby activating energy dissipation mechanisms such as MXene nanosheet fracture, extensive interfacial debonding, and fibril bridging, which collectively contributed to the highest tearing energy [50]. In contrast, when the tearing force was applied parallel to the alignment direction (0°), the crack preferentially propagated along the weak interfaces between aligned lamellae, experiencing minimal deflection and encountering fewer energy‐dissipating obstacles, resulting in the lowest tearing energy. At the oblique orientation (45°), the crack encountered a mixed‐mode, where it must navigate between propagating along weak interfaces and cutting across oriented structures, leading to observable crack deflection and an intermediate tearing energy. An Ashby‐style comparison confirmed that the PM_3‐0.18_ hydrogel occupied a unique region in the property space, simultaneously offering high tearing energy, exceptional work of fracture, and high water content, a combination rarely achieved in conventional hydrogel systems (Figure 5f; Table S2).

The hydrogel also demonstrated outstanding impact tolerance. In drop‐ball tests, bare glass and isotropic PM_3_ hydrogel‐coated glass fractured under a 35 g impact. In contrast, a thin (0.5 g) PM_3‐0.18_ hydrogel protective layer effectively shielded the glass substrate from failure even when subjected to a 60 g steel ball dropped from the same height (Figure 5g,h). This remarkable performance stemmed from the multi‐scale energy‐dissipation mechanisms inherent to the inverse nacre architecture: the stiff, aligned MXene network distributed impact stress, while the ductile PVA matrix accommodated deformation through chain slippage and reversible bond breaking, collectively enabling progressive stress relaxation and crack arrest. In summary, the inverse nacre architecture successfully unified anti‐swelling capacity, exceptional tear resistance, and high impact tolerance within a single hydrogel system. This unique integration resulted in unprecedented mechanical reliability and adaptability under extreme conditions, highlighting its significant potential for demanding applications such as biomedical implants and impact‐protective coatings.

## 3 Conclusion

We present a scalable dual‐orientation engineering strategy based on thermo‐calendering to fabricate a new family of ultra‐robust nanocomposite hydrogels. Through this process, 2D MXene nanosheets and PVA chains are unidirectionally aligned, forming an inverse nacre architecture in which a small fraction of oriented MXene nanosheets act as reinforcing “bricks” embedded within a ductile, low‐crystallinity PVA matrix serving as an energy‐dissipating “mortar”. This bioinspired structural design yields an unprecedented combination of mechanical properties while maintaining high water content, including a tensile strength of 63.48 MPa, fracture strain of 180.34%, work of fracture of 54.79 MJ/m^3^, fracture toughness of 115.98 kJ/m^2^, and water content of 63.1%, effectively overcoming classical trade‐offs among strength, work of fracture, ductility, and hydration in hydrogel materials. Remarkably, the hydrogel exhibits intrinsic crack‐insensitive behavior: propagating cracks undergo spontaneous branching and deflection, substantially extending the fracture path and activating multi‐scale energy dissipation mechanisms such as interfacial sliding, MXene pull‐out, and fibrillar bridging. This autonomous crack‐divergence capability represents an emergent self‐preservation mechanism seldom reported in synthetic hydrogels, endowing the material with exceptional fatigue resistance and flaw tolerance. Our work establishes dual‐orientation engineering as a generalizable platform for manufacturing hierarchically ordered soft materials, bridging bioinspired microstructural design with scalable production for applications in load‐bearing biomedical implants and damage‐resilient soft robotics.

## 4 Experimental Section

The experimental section is shown in the Supporting Information.

## Conflicts of Interest

The authors declare no conflicts of interest.

## Supporting information




**Supporting File**: advs75451‐sup‐0001‐SuppMat.docx.

## Data Availability

The data that support the findings of this study are available from the corresponding author upon reasonable request.
