# Supplementary material for: Architected Inverse Nacre Hydrogels With High Strength and Crack‐Insensitive Toughness
Source: Adv Sci (Weinh). 2026 May 21;13(42):e23655. doi: 10.1002/advs.202523655 (PMC13335519; doi:10.1002/advs.202523655)
Supplement: Supplementary file 1 — Supporting File: advs75451‐sup‐0001‐SuppMat.docx. [file ADVS-13-e23655-s001.docx]

Supplementary materials for

**Architected Inverse Nacre Hydrogels with High Strength and Crack-Insensitive Toughness**

Haidi Wu^1^, Qin Su^1^, Cheng Guan^1^, Biwang Pan^1^, Wenjie Hu^1^, Jun Yan^1^, Yifan Feng^1^, Longcheng Tang^2^, Xuewu Huang^1, 3^, Jiefeng Gao^1,^ *, Wancheng Gu^1,^ *

^1.^School of Chemistry and Materials, Yangzhou University, No 180, Road Siwangting, Yangzhou, Jiangsu, 225002, China

^2.^Key Laboratory of Organosilicon Chemistry and Material Technology of Ministry of Education, Hangzhou Normal University, Hangzhou 311121, China

^3.^Testing Center, Yangzhou University, Yangzhou, Jiangsu Province 225002, China

*Corresponding author: [jfgao@yzu.edu.cn](mailto:jfgao@yzu.edu.cn), seuguwancheng@163.com

**This file includes:**

Supplementary text

Figures S1 to S23

Table S1 and S2

**Supplementary Information Text**

**Experimental Section**

**Materials**

Polyvinyl alcohol (PVA, degree of polymerization of 1700 and 99% hydrolyzed), glycerol and glutaraldehyde (50 vol.%) were purchased from Aladdin. MAX phase ceramic powder (Ti_3_AlC_2_, 99%) was bought from Laizhou Kai Kai Ceramic Materials Company Ltd. Lithium fluoride (LiF, 98%) was purchased from Alfa Aesar (China) Chemical Co. Ltd. Hydrochloric acid (HCl, 36.5-38 wt.%) was acquired from Sinopharm Chemical Co. Lit. All reagents were purchased and used without further purification.

**Preparation of MXene nanosheet aqueous solution**

The preparation of MXene aqueous solution was as follows: first, 2 g of LiF and 40 mL of 9 M HCl solution were introduced into a Teflon mold. After magnetic stirring at 35 °C for 15 minutes, the LiF/HCl etching solution was prepared. Then, 2 g of MAX powder was gradually added to the aforementioned LiF/HCl solution, and the mixture was allowed to react at 35 °C for 24 h under magnetic stirring. After the etching process was completed, the resultant mixture was washed several times with deionized water and centrifuged at 3500 rpm for 5 minutes until the pH of the supernatant reached 7. Subsequently, the supernatant and precipitate were combined and subjected to ultrasonic treatment using an ultrasonic cell disruptor (JY98-IIIDN, Ningbo Xinzhi Biotechnology Co., Ltd., Ningbo, China) operating at 285 W and 20 kHz for 1 h. Finally, the mixture was centrifuged at 3500 rpm for 1 h, and the supernatant containing delaminated MXene nanosheets was collected and filtered to measure its concentration before use.

**Fabrication of PVA hydrogels**

First, a 15 wt.% PVA solution was prepared by adding a certain mass of PVA powder into the MXene aqueous solution and stirring in an oil bath at 90 °C for 5 h. The content of MXene solids in this solution was controlled to be 0 wt.%, 1 wt.%, 3 wt.%, and 5 wt.% of the PVA powder mass, respectively. After defoaming, the homogeneous PVA/MXene solution was transferred into home-made PTFE molds. Once the PVA solution cooled down, the molds containing the solution were immersed in a beaker filled with glycerol solution for solvent exchange. The entire solvent exchange process was carried out for 48 h, during which the glycerol solution was replaced every 12 h to ensure maximum replacement of water with glycerol. After 48 h of immersion, the PVA/MXene/glycerol organogels (designated as PM_x_O organogels) were obtained. These PM_x_O organogels were then cut into rectangular pieces for calendering (Laboratory desk type, BD-8818, Dongguan Baoding Precision Instrument Co., LTD, China). The calendering temperature was set at 120 °C, and the roller nip of the calender was adjusted to 0.18, 0.3, 0.4, and 0.5 mm, respectively, with an average rotation speed of 25 r/min. After 15 minutes of calendering, the organogels were pressed into thin sheets under the strong temperature-shearing field. Finally, the calendered organogel sheets were immersed in deionized water for 48 h for solvent exchange, yielding the PVA/MXene hydrogels, which were designated as PM_x-y_ hydrogels for convenience. Here, “P” and “M” are abbreviations for PVA and MXene, respectively; the subscript “x” represents the mass fraction of MXene solids relative to PVA powder, and “y” represents the roller nip of the calender. In addition, another type of PVA hydrogel (designated as PM_x_ hydrogels) was fabricated by immersing the PM_x_O organogels directly in deionized water without calendering.

**Mechanical tests**

All the uniaxial tensile tests of the PVA samples were carried out by the universal tensile tester (Instron Model 3367, USA) at room temperature. All the hydrogel samples were cut into dog bone-shaped splines (50 mm (l)× 4 mm (w)) before test and the loading rate was 50 mm/min. The work of fracture was calculated by integrating the area of stress-strain curves, and the modulus was calculated by the slope of the stress-strain at the strain ranges of 5-10%. The cyclic loading-unloading tests were conducted on the customized mechanical stretcher (FULETEST, China) at a constant rate of 50 mm/min, and the samples were first prestretched to a certain strain and then immediately unloaded.

The fracture toughness (*Γ*) of all the PVA hydrogels were calculated by the pure shear tests [1]. Both the unnotched and notched PVA hydrogel samples were cut into rectangular splines (50 mm (l) × 25 mm (w)) and the distance of clamps was about 5 mm. For the notched sample, an additional notch with the length of about 12 mm was introduced in the middle of one side of the sample. The fracture toughness of the PVA hydrogels were calculated by

|  | $\text{Γ}=\frac{\text{W}(\text{∆}\text{L}_{\text{c}})}{\text{A}}$ | (1) |
| --- | --- | --- |

where the *ΔL_c_* was the critical extension of the notched samples in which the cracks transfer to the running crack. *W*(Δ*L*_c_) is the integrated work area under the tensile force-displacement curve of the unnotched specimen from the original point to Δ*L_c_*, and *A* is the cross-section of the specimen.

**Fatigue tests**

The single-notch method was conducted to assess the fatigue resistance of the hydrogels [2, 3]. All the cyclic tensile tests were performed in the water bath to prevent water evaporation using a customized mechanical stretcher (FULETEST, China). Both the rectangular notched and unnotched samples with the length of 50 mm and width of 10 mm were used for the cyclic tensile tests, and the tensile speed was 900 mm min^-1^. For the notched samples, the initial crack length (*c*_0_) was about 1 mm. A digital camera (AF4915ZTL, Dino-Lite) was used to monitor the crack growth. The fatigue threshold (*G*) was calculated by:

|  |  | (2) |
| --- | --- | --- |

where *k* is a varying function of the tensile strain *λ*, which is obtained by the function $\text{k}\text{=3/}\sqrt{\lambda}$, *c* is the corresponding crack length after the *N*th cycle, and *W* is the strain energy density of an unnotched sample stretched to the same strain *λ*, which is calculated by:

|  | *W*(*λ*_max_, *N*)=$\int_{\text{1}}^{\text{λ}\text{max}} \text{S}\text{d}\text{λ}$ | (3) |
| --- | --- | --- |

where *λ*_max_ is the maximal applied stretch under the *N*th cycle, and *S* is the measured stress. The fatigue threshold was determined by the intercept of the linear extrapolation of the data of crack extension per cycle (d*c*/d*N*) versus *G* to the abscissa.

**Water content measurement**

The water contents of the samples were measured by comparing the weights of samples before (m_w_) and after (m_d_) water removing. The hydrogels samples were dried in an oven at 60 ℃ for 72 h to ensure that the samples were completely dried. The water content (*ϕ*_water_) was calculated by:

|  |  | (4) |
| --- | --- | --- |

**Measurement of Crystallinity**

The differential scanning calorimeter (DSC 8500, Perkin Elme, USA) was used to calculate the crystallinities of PVA hydrogels. Before the DSC tests, all the PVA hydrogels were chemically cross-linked in a cross-linking agent (5 mL glutaraldehyde, 500 μL HCl and 105 mL DI water) for 5 minutes to prevent the crosslinking of amorphous polymer chains. Then soaking the samples in DI water to remove the excess glutaraldehyde, finally the samples were placed in the oven until they were completely dried and used for DSC measurements. The heat flow of DSC showed a peak at the range of 200-250 °C, which belongs to the melting of the crystalline domains of PVA. And the enthalpy (*H_crystalline_*) of the melting of the crystalline domains per unit mass of the dry sample was calculated by integrating the crystalline peak from 200 to 250 °C. And the crystallinity of dried samples was calculated by [4]:

|  | $\text{X}_{\text{dry}}=\frac{\text{H}_{\text{crystalline}}}{\text{H}_{\text{crystalline}}^{\text{0}}}$ | (5) |
| --- | --- | --- |

*H*0 crystalline (138.6 J g^-1^) is the enthalpy for fusing 100 wt.% crystalline PVA at the equilibrium melting point *T*0 m.

and the crystallinity of the swollen PVA hydrogels can be obtained by the following equation:

|  | $\text{X}_{\text{swollen}}=\text{X}_{\text{dry}}\times\text{(1-}\text{WC}\text{)}$ | (6) |
| --- | --- | --- |

**X-ray diffraction spectra measurements**

X-ray diffractometer (XRD, Bruker AXS, Germany) was used to detect the characteristic crystallization peaks of the hydrogels. Scanning was conducted with 2θ from 5° to 80° at a scanning rate of 2° min^-1^.

**SAXS and WAXS measurements**

SAXS and WAXS measurements were conducted using the NanoSTAR (Bruker AXS, Germany) instrument equipped with X-rays with a wavelength of 0.154 nm, an operating voltage of 50 kV, and a current of 0.6 mA. The sample-to-detector distance was 1045 mm for SAXS and 60 mm for WAXS, and the exposure time was set as 600 s. The scattering range of *~~q~~* was 0.007 - 0.123 Å^-1^ for the SAXS measurements, and the WAXS profiles were collected in the 2θ range of 5° to 35°, and the scattering range was 0.21-2.45 Å^-1^. Based on the one-dimensional (1D) scattering curve of corrected scattering intensity (*Iq^2^*) *versus* scattering vector (*q*) from 2D SAXS patterns, the average distance between crystalline domains of PVA hydrogels was calculated using the following Bragg expression [5]:

|  | $\text{L}=\frac{\text{2π}}{\text{q}_{\text{max}}}$ | (7) |
| --- | --- | --- |

where q_max_ was the critical vector corresponding to the highest peak intensity. From the 1D scattering curve of intensity *versus* diffraction angle (2θ) of WAXS patterns, the average size of the crystalline domains (*D*) of PVA hydrogels was calculated based on Scherrer's equation below [6]:

|  | $\text{D}=\frac{\text{kλ}}{\text{β}\text{ cos}\theta}$ | (8) |
| --- | --- | --- |

where *k, λ, β, θ* are the dimensionless shape factor, the wavelength of X-ray diffraction, the full width at half maximum of the peak, and Bragg angle, respectively. We assume the shape of the crystalline domains of PVA hydrogel is approximately spherical, and thus *k* is set as 1.

**Scanning electronic microscopy characterizations**

Scanning electronic microscopy (SEM, ZEISS Supra55, Germany) was used to characterize the micro- and nano- structures of the hydrogels after freeze drying. All the samples were soaked in DI water for 2 d to removal the ions, and then frozen with liquid nitrogen and fractured quickly before they were freeze dried by a freeze-dryer (SCIENTZ-10N) at -80 ℃ for 48 h. All the samples were coated with gold before SEM characterization.

**Fourier transform infrared spectra measurements**

Cary610 Fourier transform infrared spectra (FTIR, Varian, USA) was used to detect the hydrogen bonding in hydrogels at the wavenumber of 400 to 4000 cm^-1^.

**Rheological measurements**

A rotational rheometer (DHR, TA Co., USA) equipped with a parallel plate (diameter of 25 mm) and a Peltier temperature control system were used to character the dynamic rheological behavior of the hydrogels. A frequency sweep was performed in the range 0.1-100 rad s^-1^ with a constant strain of 0.1%. An oscillation strain sweep (0.1 to 100%) was conducted at the constant frequency of 6.28 rad s^-1^. All rheological measurements were performed at 25 ℃.

**Statistical Analysis**

All numerical values presented in the manuscript are the mean values obtained from at least three independent measurements per sample, and the error bars in subsequent analyses represent the standard deviation (mean ± SD) of the replicate tests.

**Supplementary Figures**


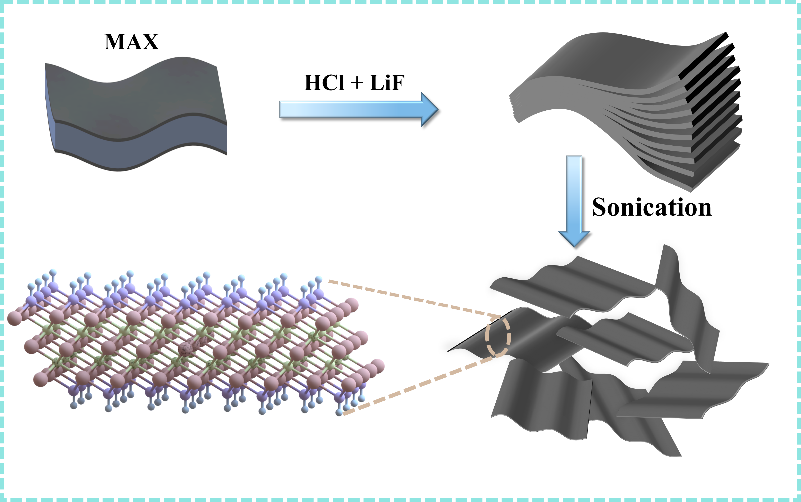


Figure S1. Schematic diagram of MXene nanosheet preparation via HF etching.


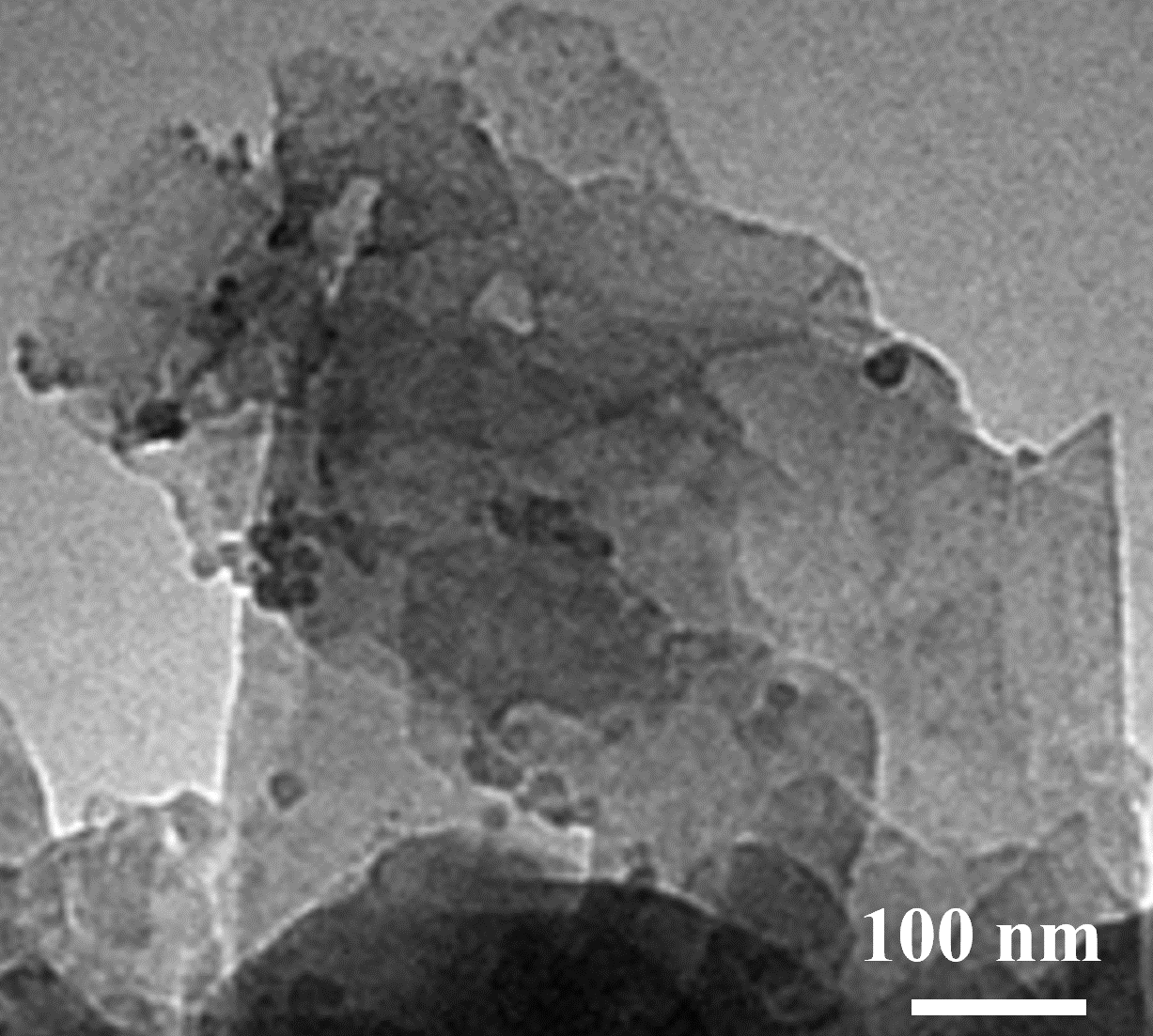


Figure S2. TEM image of delaminated MXene nanosheets.


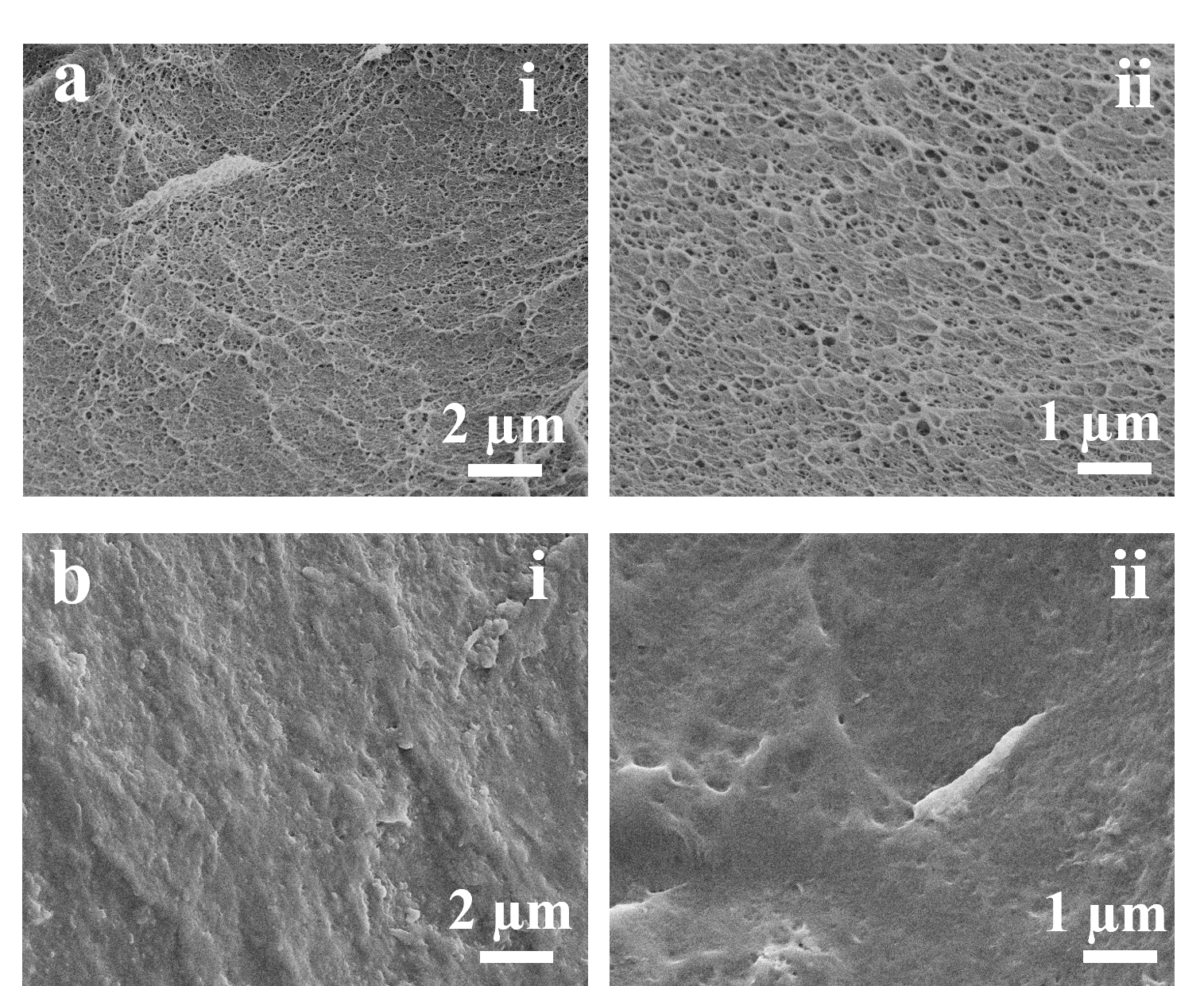


Figure S3. SEM images of the PM_0_ hydrogel (a_i_, a_ii_) and the PM_3_ hydrogel (b_i_, b_ii_).


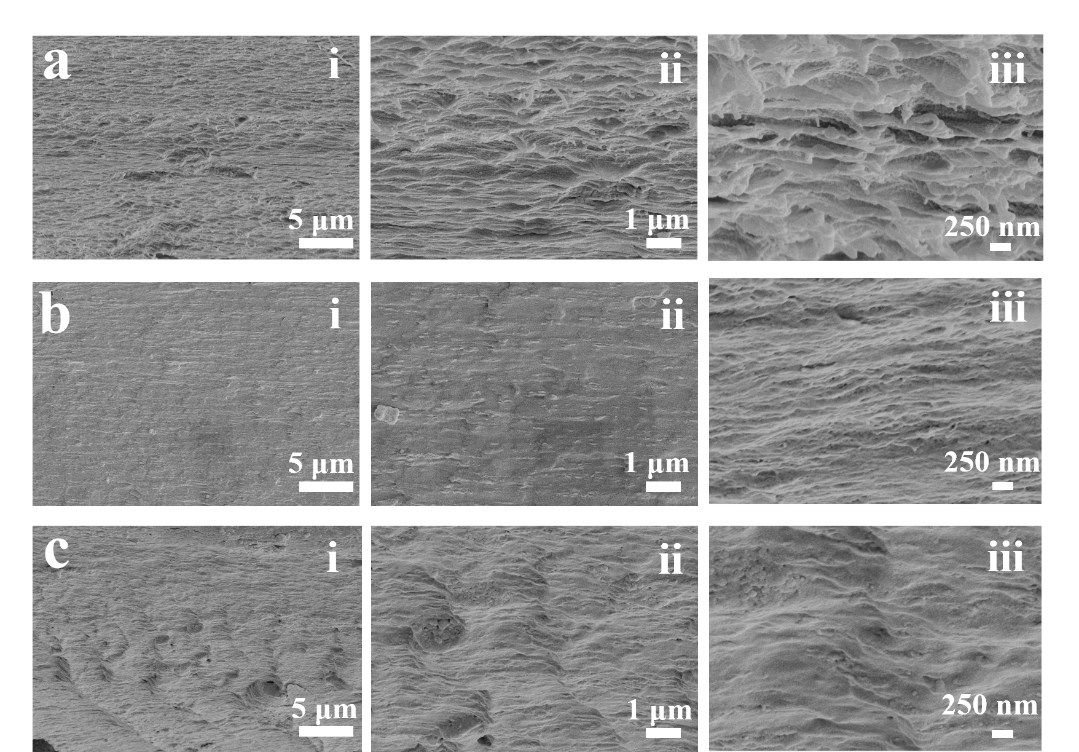


Figure S4. SEM images of the PM_0-0.18_ hydrogel (a_i_, a_ii_, a_iii_), PM_3-0.18_ hydrogel (b_i_, b_ii_, b_iii_) and PM_5-0.18_ hydrogel (c_i_, c_ii_, c_iii_).

Figure S5. DSC thermographs of different PM_1-0.18_ and PM_5-0.18_ hydrogels.

Figure S6. XRD spectra of the PM_0_ and PM_3_ hydrogels.


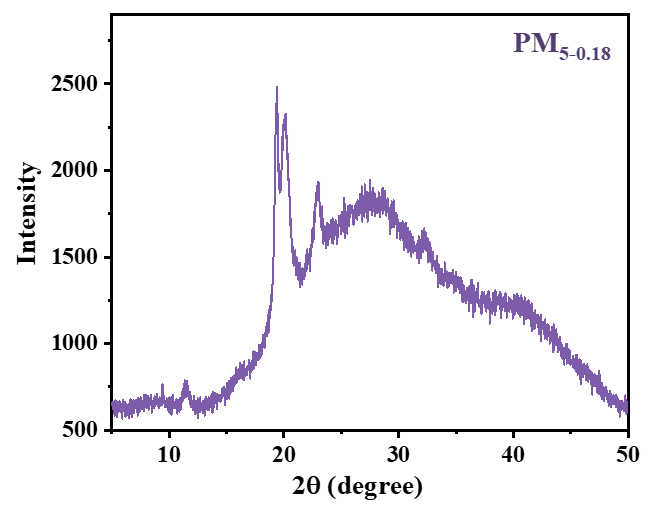


Figure S7. XRD spectra of the PM_5-0.18_ hydrogel.


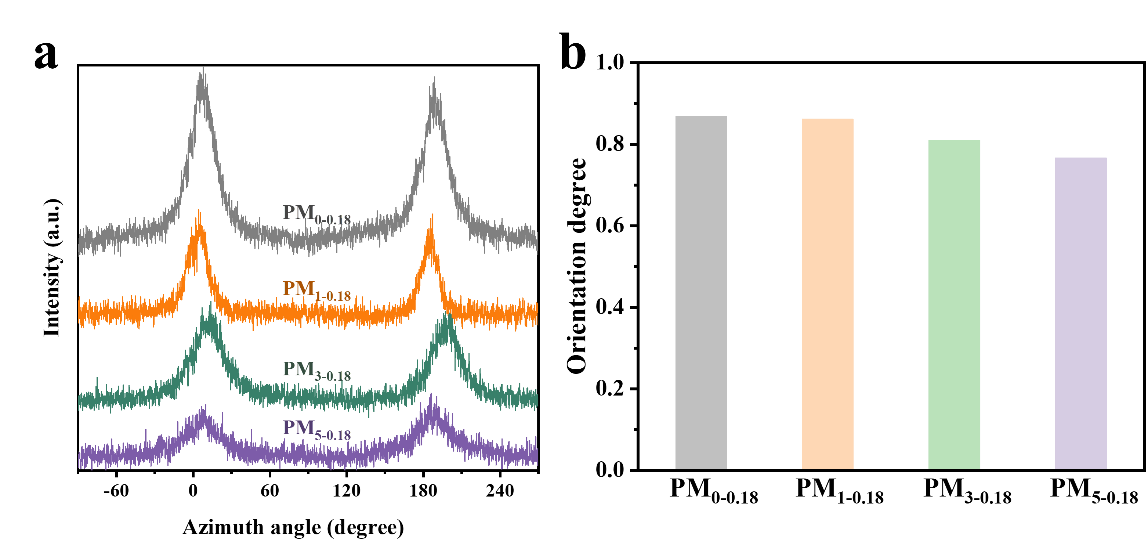


Figure S8. a) The intensity *versus* azimuth angle curves of different PM_x-0.18_ hydrogels and b) orientation degree of different PM_x-0.18_ hydrogels.

Figure S9. ATR-FTIR spectra of different PM_x-0.18_ hydrogels.


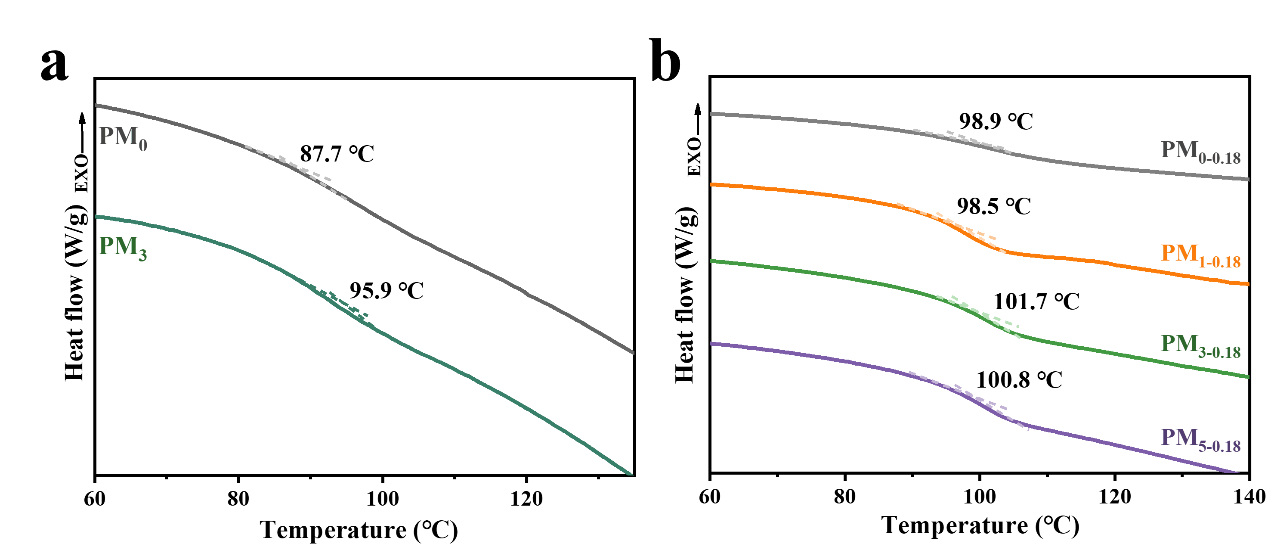


Figure S10. The glass-transition temperature (T_g_) of the PM_0_ and PM_3_ hydrogel a) and PM_X-0.18_ hydrogels b) from the DSC curves.

Figure S11. The scattering intensity *versus* scattering vector (q) curve for WAXS curve of the PM_5-0.18_ hydrogel.

Figure S12. The calculated crystal dimension of PM_x-0.18_ hydrogels by XRD.

Figure S13. The corresponding scattering intensity *versus* q curve for SAXS curve of the PM_5-0.18_ hydrogel.


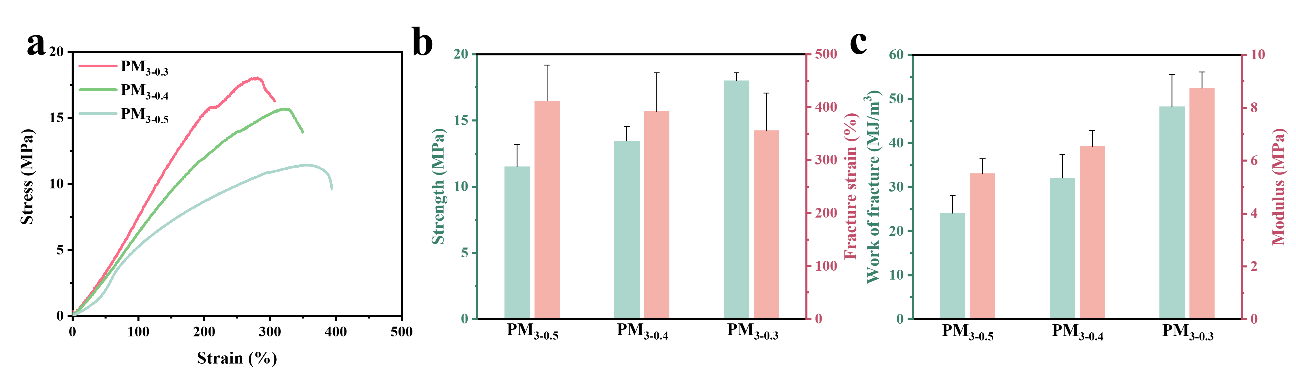


Figure S14. a) The tensile stress-strain curves of the PM_3-0.3_, PM_3-0.4_ and PM_3-0.5_ hydrogels and corresponding summary of strength, fracture strain (b), work of fracture and modulus (c) of these hydrogels.

Figure S15. The water content of the PM_x-0.18_ hydrogels.

Figure S16. The swelling kinetic curve of freeze-dried PM_3-0.18_ aerogel.


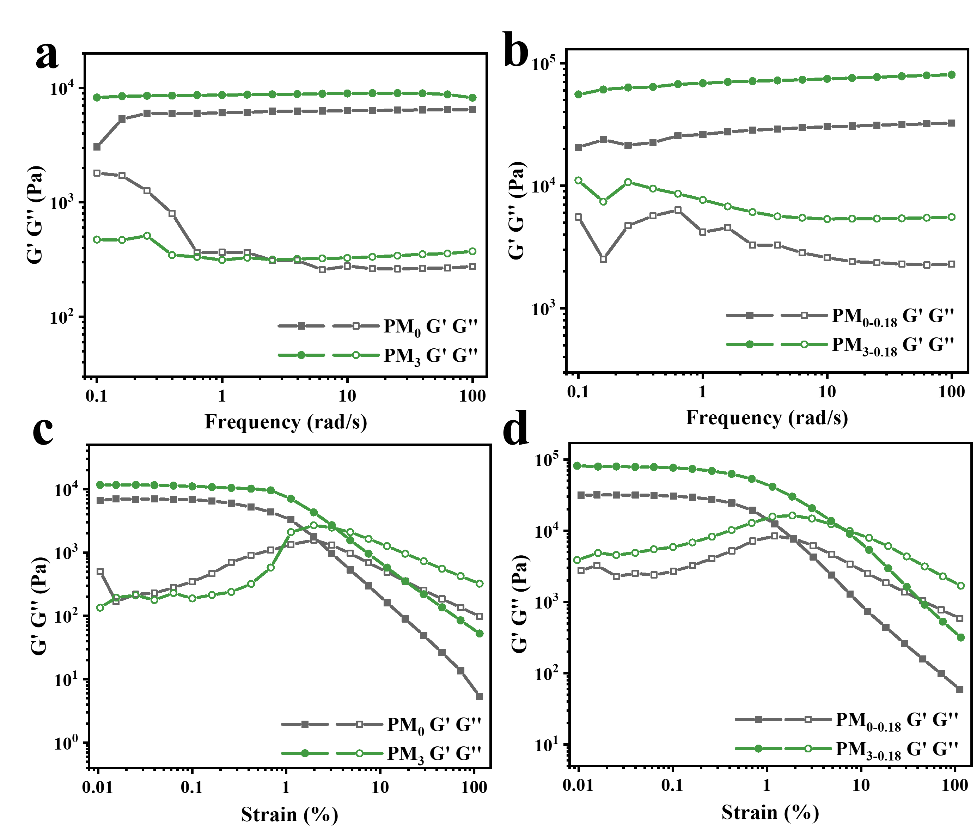


Figure S17. (a-d) The dynamic mechanical behaviors of the PM_x_ hydrogels. The variation of storage modulus (*G′*) and loss modulus (*G″*) with frequency (a-b) and strain (c-d).


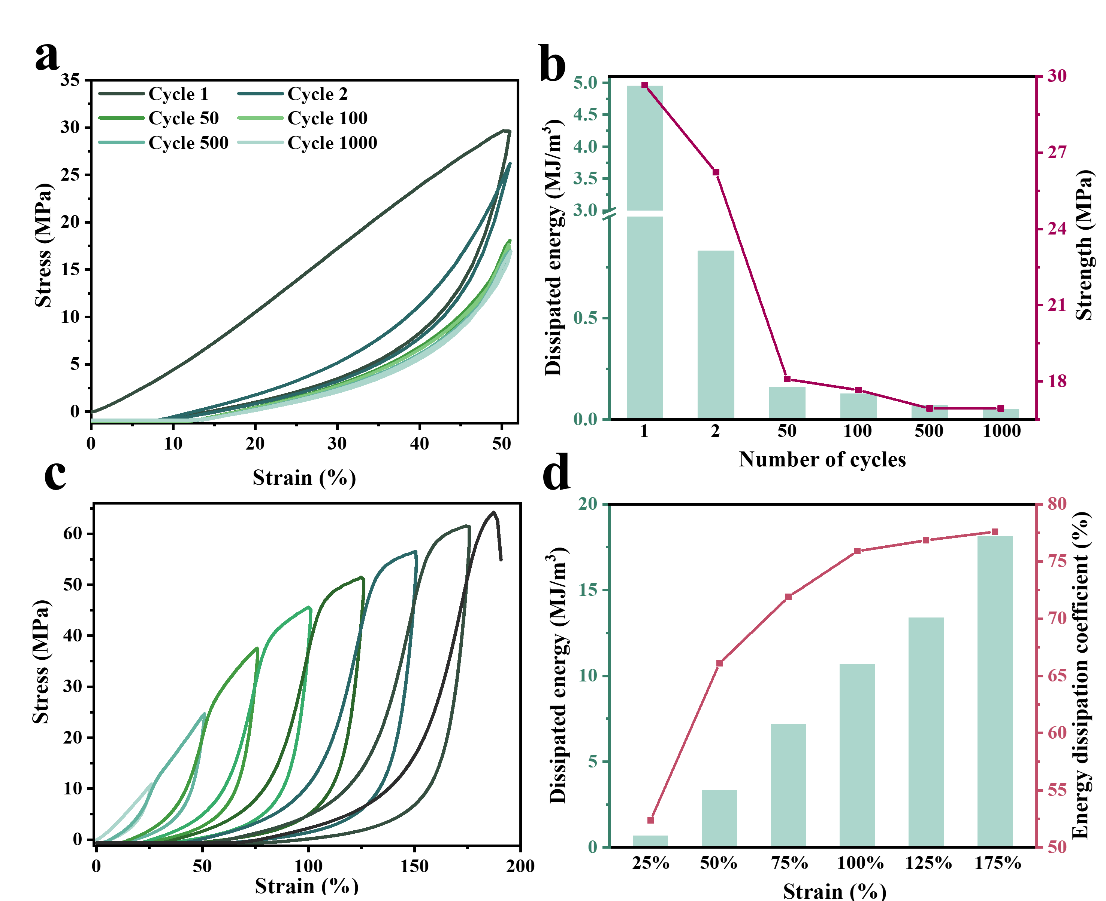


Figure S18. a) Successive tensile loading-unloading curves of the PM_3-0.18_ hydrogel for 1000 cycles at a predeterminate strain of 50% and b) the change of dissipated energy and energy dissipation coefficient during the 1000 cyclic loads; c) Tensile loading-unloading curves of the PM_3-0.18_ hydrogel under different strains of 25-175% and d) the change of dissipated energy and strength under different tensile strain.

Figure S19. Comparison plot of strength *versus* fracture toughness of the PM_3-0.18_ hydrogel with other reported anisotropic PVA hydrogel.


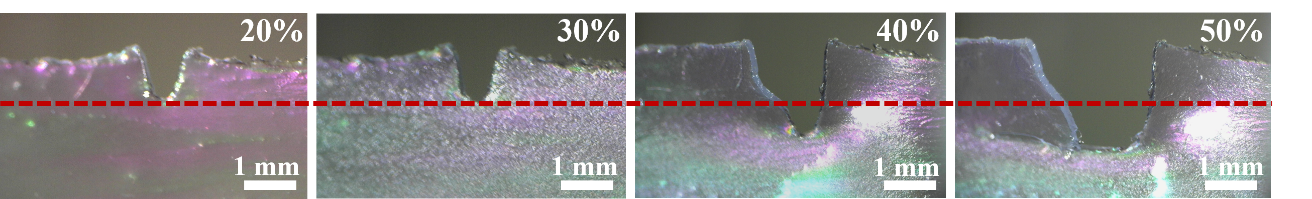


Figure S20. Photos of crack propagation paths of the PM_0-0.18_ hydrogel at different strain (Scale bar =1 mm).

Figure S21. Crack extension per cycle dc/dN *versus* applied energy release rate of the PM_0-0.18_ hydrogel.


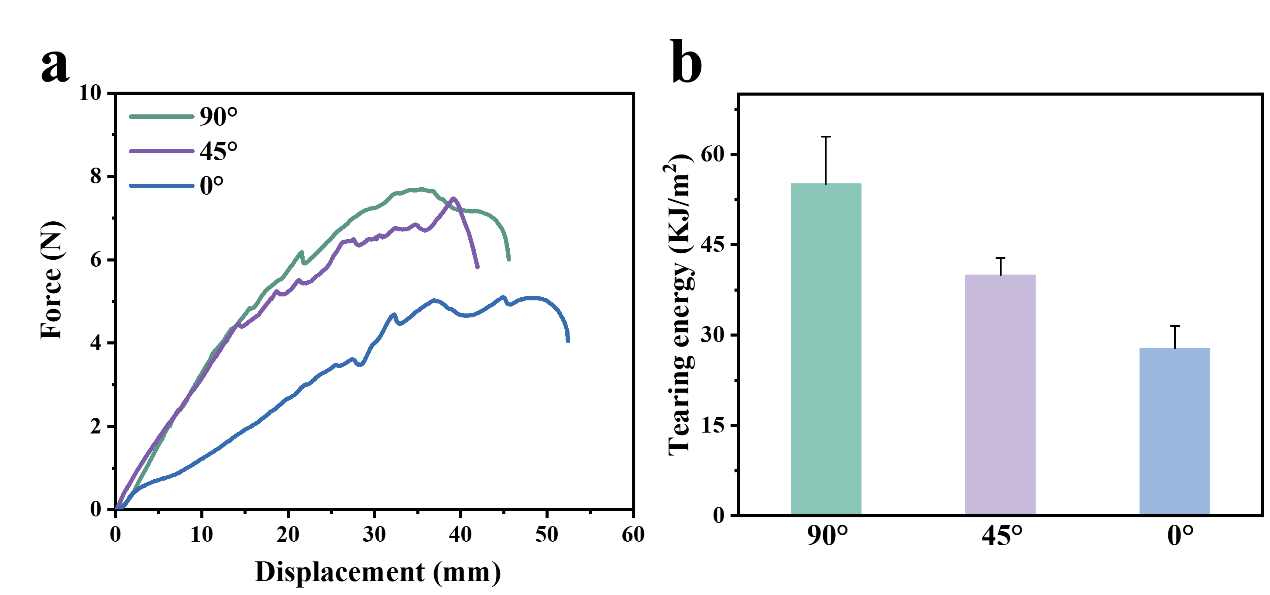


Figure S22. The force-displacement curves at various angles relative to the alignment direction of PM_3-0.18_ hydrogel (a) and corresponding tearing energy at various angles (b).


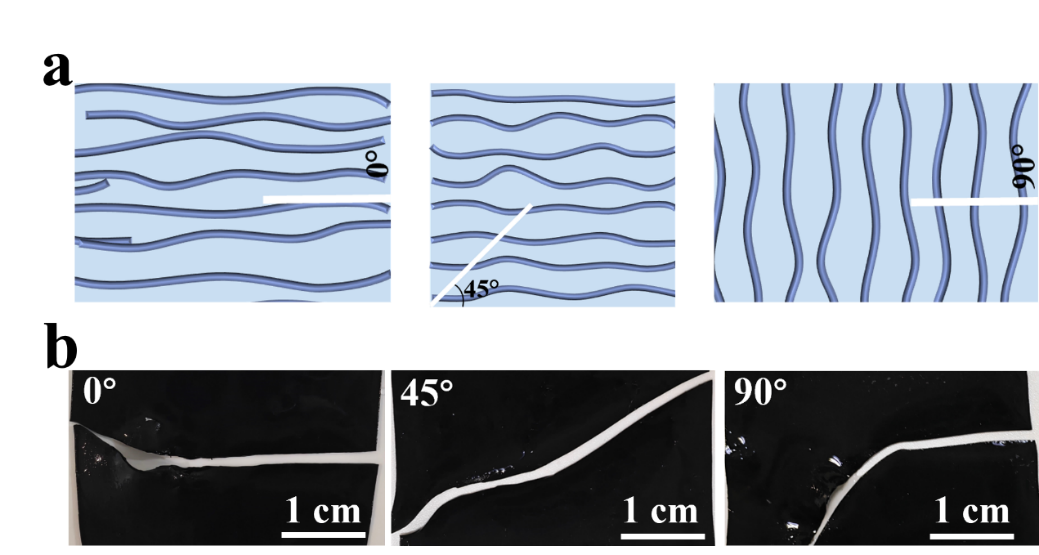


Figure S23. Schematic diagram of the tearing tests conducted at various angles relative to the alignment direction of the PM_3-0.18_ hydrogel (a), along with photographs showing the corresponding crack propagation paths under different testing angles (b).

**Table S1**: Comparison of the mechanical properties and water content of the PM_3-0.18_ hydrogel in this work with other PVA hydrogels.

| Sample | Strength  (MPa) | Fracture strain (%) | Work of fracture  (MJ/m^3^) | Fracture toughness (KJ/m^2^) | Modulus  (MPa) | Water content (%) | Ref. |
| --- | --- | --- | --- | --- | --- | --- | --- |
| PM_3-0.18_ | 63.48 | 180.34 | 54.79 | 115.98 | 43.08 | 63.14 | This  work |
| PVA/ANF | 72.1 | ~20 | N/A | 7.333 | 1114 | ~60 | [7] |
| PVA/S/S | 57.63 | N/A | ~70 | N/A | 16.87 | 40.37 | [8] |
| HHPC | 55.3 | ~3300 | N/A | 552.7 | 6.8 | 46.5 | [9] |
| AH-200 | 35.34 | 122 | 18.27 | 106.18 | 46.37 | 57 | [10] |
| PVA/HA | 23.5 | 2900 | 210 | 170 | N/A | ~70 | [3] |
| PAHL | 24.15 | N/A | 15.68 | N/A | 18.56 | 74.4 | [11] |
| PVA/TA | 19.3 | N/A | 32.1 | 38.4 | 35.6 | 53.4 | [12] |
| PVA/M/H | 16.57 | 464 | 39.23 | N/A | 8.58 | 57 | [13] |
| PVA/PVP | 6.3 | N/A | 69.8 | 67.2 | 1.7 | ~83 | [14] |
| BNCH | 10.22 | 664 | 41 | N/A | 3.8 | 54 | [15] |
| AFH_24_ | 14 | N/A | 154 | 153 | N/A | 58 | [16] |
| PVA/GO | 8.8 | N/A | N/A | N/A | ~9 | 82.3 | [17] |
| PVA/CNT | 4.5 | 440 | N/A | N/A | N/A | 79.5 | [18] |
| PVA/LS/S | 23 | 350 | 47 | 42 | 17 | 38.39 | [19] |
| PVA/K/ANFF | 17.9 | 66.3 | 11.8 | N/A | 181.2 | N/A | [20] |
| PTW | 5.4 | 730 | 18.5 | 134.9 | 1.2 | 37.5 | [21] |
| PP | 3.71 | 505 | 9.86 | N/A | 1.1 | 72.5 | [22] |

**Table S2**: Comparison of the mechanical properties and tearing energy of the PM_3-0.18_ hydrogel in this work with other hydrogels.

| Sample | Strength  (MPa) | Fracture strain (%) | Work of fracture  (MJ/m^3^) | Tearing energy  (KJ/m^2^) | Water content (%) | Ref. |
| --- | --- | --- | --- | --- | --- | --- |
| PM_3-0.18_ | 63.48 | 180.34 | 54.79 | 55.11 | 63.14 | This  work |
| SRC | 18.5 | 105.5 | 14.7 | 53.312 | 52 | [23] |
| PIMA/Na_2_SO_4_ | 12.65 | ~170 | 19.6 | 34 | N/A | [24] |
| WA/SiO_2_ | 5.37 | 661 | 18.75 | 29.14 | N/A | [25] |
| WAN_3_Si | 5.11 | 577 | 17.16 | 18.04 | N/A |  |
| WE/PVA_20_ | 7.8 | ~640 | 29.48 | 18.8 | N/A | [26] |
| PCL/PNAGA | 2.73 | N/A | 29.41 | 17 | ~64 | [27] |
| PNASC/PCBAA | 4.34 | N/A | 25.49 | 12.7 | 45.8 | [28] |
| PVA/urea/SiO_2_ | 8.1 | N/A | 44.45 | 1.44 | ~65 | [29] |

**Reference**

1. J. Ma, X. Zhang, D. Yin, Y. Cai, Z. Shen, Z. Sheng, J. Bai, S. Qu, S. Zhu, Z. Jia, "Designing ultratough single-network hydrogels with centimeter-scale fractocohesive lengths via inelastic crack blunting," *Adv. Mater.* (2024): 2311795, <https://doi.org/10.1002/adma.202311795>

2. S. Lin, X. Liu, J. Liu, H. Yuk, H.-C. Loh, G. A. Parada, C. Settens, J. Song, A. Masic, G. H. McKinley, X. Zhao, "Anti-fatigue-fracture hydrogels," *Sci. Adv.* eaau8528, https://doi.org/10.1126/sciadv.aau8528

3. M. Hua, S. Wu, Y. Ma, Y. Zhao, Z. Chen, I. Frenkel, J. Strzalka, H. Zhou, X. Zhu, X. He, "Strong tough hydrogels via the synergy of freeze-casting and salting out," *Nature* (2021): 594, https://doi.org/10.1038/s41586-021-03212-z

4. Y. Wu, Y. Zhang, H. Wu, J. Wen, S. Zhang, W. Xing, H. Zhang, H. Xue, J. Gao, Y. Mai, "Solvent-exchange-assisted wet annealing: A new strategy for superstrong, tough, stretchable, and anti-fatigue hydrogels," *Adv. Mater.* (2023): 2210624, <https://doi.org/10.1002/adma.202210624>

5. X. Liang, G. Chen, I. M. Lei, P. Zhang, Z. Wang, X. Chen, M. Lu, J. Zhang, Z. Wang, T. Sun, Y. Lan, J. Liu, "Impact-resistant hydrogels by harnessing 2d hierarchical structures," *Adv. Mater.* (2023): 2207587, <https://doi.org/10.1002/adma.202207587>

6. W. Li, L. Li, Z. Liu, S. Zheng, Q. Li, F. Yan, "Supramolecular ionogels tougher than metals," *Adv. Mater.* (2023): 2301383, <https://doi.org/10.1002/adma.202301383>

7. M. Sun, H. Li, Y. Hou, N. Huang, X. Xia, H. Zhu, Q. Xu, Y. Lin, L. Xu, "Multifunctional tendon-mimetic hydrogels," *Sci. Adv.* eade6973, https://doi.org/10.1126/sciadv.ade6973

8. Y. Chen, X. Sun, L. Luo, H. Li, N. Chen, C. Yan, X. Liu, J. Li, M. Qin, J. Qin, P. Cheng, "Super strong and tough pva hydrogel fibers based on an ordered-to-disordered structural construction strategy targeting artificial ligaments," *Adv. Funct. Mater.* (2025): 2415737, <https://doi.org/10.1002/adfm.202415737>

9. Z. Xu, H. Chen, H.-B. Yang, X. Yao, H. Qin, H.-P. Cong, S.-H. Yu, "Hierarchically aligned heterogeneous core-sheath hydrogels," *Nat. Commun.* (2025): 400, 10.1038/s41467-024-55677-x

10. H. Li, Y. Zhang, H. Wu, Z. Liu, C. Guan, J. Zhang, J. Chen, S. He, X. Huang, W. Gu, Y. W. Mai, J. Gao, "Strong and fatigue-resistant hydrogels via poor solvent evaporation assisted hot-stretching for tendon repair," *Adv. Sci.* (2025): 2503697, <https://doi.org/10.1002/advs.202503697>

11. Y. Yu, Z. Liu, Q. Zhao, "Inorganic ionic oligomers induced organic-inorganic synergistic toughening enabling mechanical robust and recyclable nanocomposite hydrogels," *Adv. Funct. Mater.* (2023): 2213699, <https://doi.org/10.1002/adfm.202213699>

12. C. Luo, M. Huang, X. Sun, N. Wei, H. Shi, H. Li, M. Lin, J. Sun, "Super-strong, nonswellable, and biocompatible hydrogels inspired by human tendons," *ACS Appl. Mater. Interfaces* (2022): 2638, https://doi.org/10.1021/acsami.1c23102

13. B. Guo, Y. Wu, S. He, C. Wang, M. Yao, Q. Yu, X. Wu, C. Yu, M. Liu, L. Liang, Z. Zhao, Y. Qiu, F. Yao, H. Zhang, J. Li, "Anisotropic and super-strong conductive hydrogels enabled by mechanical stretching combined with the hofmeister effect," *J. Mater. Chem. A* (2023): 8038, https://doi.org/10.1039/D2TA09973J

14. Y. Wang, J. Li, N. Muhammad, Z. Wang, D. Wu, "Hierarchical networks of anisotropic hydrogels based on cross-linked poly(vinyl alcohol)/poly(vinylpyrrolidone)," *Polymer* (2022): 124920, <https://doi.org/10.1016/j.polymer.2022.124920>

15. Y. Huang, X. Zhang, T. Zhu, Y. Wang, N. Hu, Z. Ren, X. Yu, D. H. Nguyen, C. Zhang, T. Liu, "Aligned porous and anisotropic nanocomposite hydrogel with high mechanical strength and superior puncture resistance by reactive freeze-casting," *Chem. Mater.* (2023): 5809, https://doi.org/10.1021/acs.chemmater.3c00368

16. S. Zhu, S. Wang, Y. Huang, Q. Tang, T. Fu, R. Su, C. Fan, S. Xia, P. S. Lee, Y. Lin, "Bioinspired structural hydrogels with highly ordered hierarchical orientations by flow-induced alignment of nanofibrils," *Nat. Commun.* (2024): 118, https://doi.org/10.1038/s41467-023-44481-8

17. X. Liang, G. Chen, S. Lin, J. Zhang, L. Wang, P. Zhang, Y. Lan, J. Liu, "Bioinspired 2d isotropically fatigue-resistant hydrogels," *Adv. Mater.* (2022): 2107106, <https://doi.org/10.1002/adma.202107106>

18. S. Han, Q. Wu, J. Zhu, J. Zhang, A. Chen, S. Su, J. Liu, J. Huang, X. Yang, L. Guan, "Tough hydrogel with high water content and ordered fibrous structures as an artificial human ligament," *Mater. Horizons* (2023): https://doi.org/10.1039/D2MH01299E

19. X. Pan, X. Li, Z. Wang, Y. Ni, Q. Wang, "Nanolignin-facilitated robust hydrogels," *ACS Nano* (2024): 24095, https://doi.org/10.1021/acsnano.4c04078

20. H. J. Kim, H. Kim, Y. H. Choi, E. S. Lee, Y. H. Kim, G.-H. Lee, H. G. Chae, Y. Eom, "Rapid fabrication of tendon-inspired ultrastrong, water-rich hydrogel fibers: Synergistic engineering of cyano-p-aramid nanofibers and poly(vinyl alcohol)," *ACS Nano* (2025): 8316, https://doi.org/10.1021/acsnano.4c18686

21. X.-J. Zha, B. Zhang, Z.-C. Cheng, S. Zhang, J.-H. Pu, J.-G. Huang, W. Yang, "Solvent-exchange triggered hydrogen bond activation strategy toward self-adaptive strong and tough organohydrogel artificial muscle," *Chem. Eng. J.* (2023): 146548, <https://doi.org/10.1016/j.cej.2023.146548>

22. N. Li, Q. Yu, S. Duan, Y. Du, X. Shi, X. Li, T. Jiao, Z. Qin, X. He, "Anti-swelling, high-strength, anisotropic conductive hydrogel with excellent biocompatibility for implantable electronic tendon," *Adv. Funct. Mater.* (2024): 2309500, <https://doi.org/10.1002/adfm.202309500>

23. C. Xiang, L. Lei, H. Ning, N. Hu, A. Li, Y. Liu, F. Liu, R. Zou, J. Wen, X. Wu, L. Zhang, L. Wu, Y. Gong, J. Liu, "A self-reinforced tough and multifunctional polyvinyl alcohol fabric composite hydrogel," *Compos. Sci. Technol.* (2023): 110212, <https://doi.org/10.1016/j.compscitech.2023.110212>

24. B. Tang, J. Hu, Z. Zhao, S. Li, H. Lv, X. Yang, "Puncture-resistant hydrogels with high mechanical performance achieved by the supersaturated salt," *Mater. Horizons* (2025): 4229, https://doi.org/10.1039/D4MH01862A

25. X. Tan, W. Deng, S. Feng, T. Xu, Y. Wang, J. Zhao, J. Rong, "Pva/silica/mucin hydrogel with high toughness and low friction," *Polymer* (2025): 128543, <https://doi.org/10.1016/j.polymer.2025.128543>

26. J. Wu, J. Zhang, Y. Chen, W. Ji, Q. Wu, L. Guan, "Fatigue-resistant hydrogels with programmable crystalline domain crosslinking enabled by coordinated thermal-solvent strategy," *J. Mater. Chem. A* (2025): 29006, https://doi.org/10.1039/D5TA05010C

27. Q. Zhang, Z. Xu, X. Zhang, C. Liu, R. Yang, Y. Sun, Y. Zhang, W. Liu, "3d printed high-strength supramolecular polymer hydrogel-cushioned radially and circumferentially oriented meniscus substitute," *Adv. Funct. Mater.* (2022): 2200360, <https://doi.org/10.1002/adfm.202200360>

28. C. Fan, Z. Xu, T. Wu, C. Cui, Y. Liu, B. Liu, J. Yang, W. Liu, "3d printing of lubricative stiff supramolecular polymer hydrogels for meniscus replacement," *Biomaterials Science* (2021): 5116, https://doi.org/10.1039/D1BM00836F

29. X. Tan, C. Liang, S. Bai, P. Lan, Y. Ren, J. Zhao, J. Rong, "Pva/silica hybrid hydrogel with ultra-high strength and toughness," *Journal of Materials Science* (2024): 6916, https://doi.org/10.1007/s10853-023-09061-7
